# Susceptibility to Infections During Acute Liver Injury Depends on Transient Disruption of Liver Macrophage Niche

**DOI:** 10.3389/fimmu.2022.892114

**Published:** 2022-07-28

**Authors:** Mateus Eustáquio Lopes, Brenda Naemi Nakagaki, Matheus Silvério Mattos, Gabriel Henrique Campolina-Silva, Raquel de Oliveira Meira, Pierre Henrique de Menezes Paixão, André Gustavo Oliveira, Lucas D. Faustino, Ricardo Gonçalves, Gustavo Batista Menezes

**Affiliations:** ^1^ Center for Gastrointestinal Biology, Departamento de Morfologia, Instituto de Ciências Biológicas, Universidade Federal de Minas Gerais (UFMG), Belo Horizonte, Brazil; ^2^ Department of Biochemistry and Immunology, Instituto de Ciências Biológicas, Universidade Federal de Minas Gerais (UFMG), Belo Horizonte, Brazil; ^3^ Centre de Recherche du Centre Hospitalier Universitaire de Québec, Université Laval, Québec, QC, Canada; ^4^ Macrophage and Monocyte Biology Laboratory, Department of Pathology, Instituto de Ciências Biológicas, Universidade Federal de Minas Gerais (UFMG), Belo Horizonte, Brazil; ^5^ Department of Physiology and Biophysics, Instituto de Ciências Biológicas, Universidade Federal de Minas Gerais (UFMG), Belo Horizonte, Brazil; ^6^ Center for Immunology and Inflammatory Diseases, Division of Rheumatology, Allergy and Immunology, Massachusetts General Hospital, Harvard Medical School, Boston, MA, United States

**Keywords:** Kupffer cell, Macrophage niche, Acute liver injury, Inflammation, Cell therapy, Systemic infection

## Abstract

Kupffer cells are the primary liver resident immune cell responsible for the liver firewall function, including clearance of bacterial infection from the circulation, as they are strategically positioned inside the liver sinusoid with intimate contact with the blood. Disruption in the tissue-resident macrophage niche, such as in Kupffer cells, can lead to a window of susceptibility to systemic infections, which represents a significant cause of mortality in patients with acetaminophen (APAP) overdose-induced acute liver injury (ALI). However, how Kupffer cell niche disruption increases susceptibility to systemic infections in ALI is not fully understood. Using a mouse model of ALI induced by APAP overdose, we found that Kupffer cells upregulated the apoptotic cell death program and were markedly reduced in the necrotic areas during the early stages of ALI, opening the niche for the infiltration of neutrophils and monocyte subsets. In addition, during the resolution phase of ALI, the remaining tissue macrophages with a Kupffer cell morphology were observed forming replicating cell clusters closer to necrotic areas devoid of Kupffer cells. Interestingly, mice with APAP-induced liver injury were still susceptible to infections despite the dual cellular input of circulating monocytes and proliferation of remaining Kupffer cells in the damaged liver. Therapy with bone marrow-derived macrophages (BMDM) was shown to be effective in occupying the niche devoid of Kupffer cells following APAP-induced ALI. The rapid BMDM migration to the liver and their positioning within necrotic areas enhanced the healing of the tissue and restored the liver firewall function after BMDM therapy. Therefore, we showed that disruption in the Kupffer cell niche and its impaired function during acute liver injury are key factors for the susceptibility to systemic bacterial infections. In addition, modulation of the liver macrophage niche was shown to be a promising therapeutic strategy for liver injuries that reduce the Kupffer cell number and compromise the organ function.

## Introduction

The liver is a complex organ that plays a pivotal role in body physiology. Liver resident macrophages, named Kupffer cells (KCs), are an essential liver component (1, 2), comprising one of the largest tissue-resident macrophage populations in adult mammals (3–5). These cells originate from erythro-myeloid progenitor from yolk-sac, migrate to the fetal liver during embryogenesis where they further differentiate, and become mature macrophages after birth (6–8). During adulthood, hepatic tissue factors, such as macrophage colony-stimulating factor (CSF-1), TGF-β, and desmosterol, maintain the KCs niche within the liver (9, 10). These tissue factors are responsible for preserving the population pool by self-renewal with minimal input from the circulating monocytes (11). In addition, the strategic positioning of KCs inside the liver sinusoids, promoting direct contact with the circulation, is a fundamental feature for iron recycling, lipid metabolism, debris removal, as well as phagocytosis and elimination of circulating or gut-derived pathogens (1). However, such KCs niche might be disrupted in several liver inflammatory conditions, and those functions might be ultimately lost (12–15).

Acetaminophen (APAP) overdose is the leading cause of acute liver injury (ALI) in humans (16, 17). The hepatotoxic effects of APAP bioactivation involve the accumulation of N-acetyl-p-benzoquinone imine (NAPQI) inside hepatocytes, which drive a harmful intracellular oxidative stress response, leading to necrotic hepatocyte death and release of damage-associated patterns (DAMPs), such as DNA, histones, and HMGB1 (18). APAP overdose induces a robust inflammatory response in the liver dominated by an influx of neutrophils and subtypes of myeloid cells. Neutrophils are the predominant immune cell type in the first hours and have been described to be directly involved in the increase in tissue damage (18, 19). Also, ALI leads to a massive reduction in the KCs population, causing a major impairment in the liver firewall function (20, 21). Concomitantly, circulating monocytes are recruited to the liver in a time-dependent manner. Infiltration of the pro-inflammatory monocyte subset, characterized by the expression of the glycoprotein Ly6C, is thought to amplify ALI in early stages (22), whereas - at a later time point - a pro-resolutive monocyte subset (Ly6C^-^) is thought to restrains the inflammatory response, which is a crucial step to in restoring liver homeostasis (20, 23). Although the recruitment of inflammatory cells is critical for tissue repair, uncontrolled inflammation can evolve to a systemic inflammatory response and, ultimately, acute liver failure (ALF) (24). Such disease outcomes are problematic as therapeutic options for APAP-induced liver injury are very limited. Administration of N-acetylcysteine (NAC) is the only treatment available, but its efficacy is limited to the first 12h after APAP overdose (16, 24). As an aggravating factor for the lack of treatment, patients with ALF are often affected by systemic infections, which result in increased mortality and morbidity rates (24–26).

The reduction in the KC population and their functional impairment during ALI is a strong candidate for the increased mortality rates in these patients (20, 21, 27). Despite the rapid recruitment of monocytes in the acute phase of the lesion, several immune-related liver functions are severely impaired (27). In addition, the dynamics of the KC niche and how these cells are recovered after organ damage are still unclear. It is well accepted that infiltrating monocytes can differentiate into liver resident macrophages in the resolution phase of liver injuries (12, 28). On the other hand, the remaining KCs may also contribute to a new macrophage pool through proliferation in the resolution phase (20). Also, the program for the liver macrophage niche replacement seems to depend on the type of inflammation, and the extension of reduction in the KC population, impacting ultimately the kinetics of the organ return to homeostasis (29). Considering that the major part of these risks arises from a malfunction of resident macrophages, therapy with bone marrow-derived macrophages (BMDM) emerges as a potential therapy for ALI (30). In fact, it has been demonstrated that intravenous injection of BMDM rescues liver fibrosis, promoting a proper healing response after chronic injuries (31). Also, it has been shown that therapy with BMDM that were polarized to an alternative activation profile accelerates and improves the resolution phase of ALI and reduces liver failure. In this case, newly transferred macrophages displayed high phagocytic capacity and released pro-regenerative mediators (32), although the effects of BMDM therapy on the acute phase of ALI or ALF remain elusive.

In this work, we hypothesized that transient disruption in the Kupffer cell niche during ALI is responsible for the susceptibility to systemic infections. We revealed that APAP-mediated liver damage led to a massive KCs apoptosis and death, which triggered time-dependent recruitment of monocyte subsets that transiently occupied the empty KC niche. We showed that proliferation of KCs and infiltration of monocyte-derived macrophages restored the hepatic macrophage population following ALI and remained in the liver during the organ healing phase. Notably, during KC niche disruption and transient liver macrophage recovery create an open window for systemic infections, shown by increased circulating bacteria after infection. Ultimately, by employing a BMDM therapy to accelerate hepatic macrophage niche occupation, we found that transferred BMDM were strategically positioned in the necrotic areas of the liver, where KCs were absent during ALI. These engrafted BMDM partially restored the liver bacterial clearance ability, which was lost in the acute phase of the acute liver injury model.

## Materials and Methods

### Mice

C57BL/6J female mice aged 8-10 weeks were acquired from Centro de Bioterismo do Instituto de Ciências Biológicas – UFMG (CEBIO/UFMG). CX3CR1^+/GFP^+^^CCR2^+/rfp^ 8-10 weeks of old female mice were obtained from the animal facility of Laboratório de Imunofarmacologia - ICB/UFMG crossing the CX3CR1^GFP^+^/GFP^+^^ (B6.129P(Cg)-*Cx3cr1^tmLitt^*/J) mice with CCR2^rfp/rfp^ (B6.129S4-*Ccr2^tm1lfc^*/J). LysM^+/cre^Rosa26^+/tdTomato^ female mice of 4-6 weeks were obtained from the animal facility of Laboratório de Imunofarmacologia - ICB/UFMG crossing the LysM^cre/cre^ (B6.129P2-*Lys2^tm1(cre)lfo^*/J) with the Rosa26^tdTomato/tdTomato^ lineage (B6.Cg-*Gt(Rosa)26Sor^tm14(CAG-tdTomato)Hze^*/J). Mice were kept in the animal facility of Laboratório de Imunofarmacologia - ICB/UFMG using mouse microisolator (Alesco, Monte Mor, SP, BR) under controlled conditions of temperature, 23°C, light/dark cycle of 12/12h with sterile water and food (Nuvilab, Curitiba, PR, BR) *ad libitum*. All procedures were approved by CEUA/UFMG 377/2016 and 076/2020 protocols.

### Acute Liver Injury Model Induced by Acetaminophen Overdose

Mice were fasted for 15 hours before oral gavage of acetaminophen (APAP, Sigma-Aldrich-, St. Louis, MO, USA), 600mg/kg, or 0.9% saline solution (33). APAP was diluted in a pre-warmed saline solution. ALI kinetics was observed at 12, 24, 72 hours, 7, and 15 days post oral gavage to perform the following assays. The survival rate was checked in a 6 hours intervals from starting point to 15 days.

### Histopathologic Analysis

Liver fragments were collected for histology. The tissue was fixed in 4% paraformaldehyde in phosphate buffer saline (PBS, Sigma-Aldrich), then dehydrated in alcohol and embedded in paraffin. Sections of 4µm thicknesses were stained for hematoxylin and eosin (H&E). Images were acquired using a 4x objective lens in a Nikon Ti A1R microscope (Nikon, Shinagawa, Tokyo, Japan).

### Alanine Aminotransferase (ALT) Assay

Serum alanine aminotransferase (ALT) activity was performed using a kinetic test (Bioclin, Belo Horizonte, MG, Brazil). Serum was acquired after blood harvest, followed by 1500x *g* 10 minutes centrifugation. Briefly, the enzymatic assay was performed in 96 well-plate (Corning, New York, NY, USA) where pure serum or sequentially dilute was added. Substrates and coenzymes provided and specified in the kit were added at 37°C after 1 min plate was read in a spectrophotometer (Versamax, Molecular Devices, San Jose, CA, USA) at 37°C every 1 min for 4 min.

### Indocyanine Green Depuration Assay

The Indocyanine green (ICG, Sigma-Aldrich) assay measures the liver depuration capacity (34). Mice received a single intravenous injection of ICG (20mg/kg); 20 min later, blood samples were collected, and the serum was obtained following centrifugation 1500x *g* 10 minutes. ICG concentration was determined by spectrophotometry (Molecular Devices) at 800nm.

### Liver Non-Parenchymal Cells Isolation

To isolate non-parenchymal cells (NPC), first, mice were anesthetized subcutaneously with a mixture of ketamine (60mg/kg, Syntec, Tamboré, SP, BR) and xylazine (15mg/kg, Syntec) then the blood was collected by exsanguination *via* the femoral vein. The peritoneal cavity was exposed, and the liver was harvested and fragmented into small pieces followed by enzymatic digestion with collagenase VII (1mg/ml, Sigma-Aldrich) in RPMI-1640 medium (Gibco, Billings, MT, USA) for 60 minutes in constant agitation at 37°C. Next, liver homogenates were filtered using a 70µm cell strainer to remove undigested tissue. Then, a series of differential centrifugation, 400x *g* 5 min, 60x *g* 3 min, 400x *g* 5* min*, was performed. Finally, red blood cells were lysate Ammonium Chloride solution (ACK) for a clean NPC suspension. The cell suspension was counted, and 10^6^ cells were used for the Flow Cytometry assay.

### Flow Cytometry

NPCs suspension, when applied, was first stained for viability with Fixable Viability Stain 510, 1µL per million cells, for 15 minutes at room temperature. Then, the staining was performed for 30 min at 4°C using Fc block Fc-γ III/II CD16/32 (clone 2.4G2; BD Biosciences, Franklin Lakes, New Jersey, USA), anti-CD45 (clone 30-F11, BD Biosciences), anti-F4/80 (clone T45-2342, BD Biosciences), anti-CD11b (clone M1/70, BD Biosciences), anti-Ly6C (clone AL-21, BD Biosciences), anti-Ly6G (clone 1A8, BD Biosciences). Anti-Clec4F was not used to detect Kupffer cells because it was not reliable in intravital confocal microscopy. For cell death program staining, Annexin V (Biolegend, San Diego, CA, USA) and Propidium Iodide (Life Technologies, Carlsbad, CA, USA) staining was performed after surface staining as previously described (35). Finally, flow cytometry was performed using CytoFlex (Beckman Coulter, Indianapolis, IN, USA) or Accuri™ C6 (BD Biosciences).

### *Escherichia Coli* Cultivation

The *Escherichia coli* transfected with a plasmid containing green fluorescent protein (GFP^+^, ATCC^®^25922GFP^+^) and Ampicillin dependent promoter was gently provided by Dr. Shirong Liu (Harvard Medical School, Boston, USA). First, the bacteria culture was performed using Luria-Bertani broth (Sigma-Aldrich) containing Ampicillin for 12 hours under agitation at 37°C. Next, the culture was washed at 1500x *g* for 15 min. Finally, bacterial amounts were determined by optical density at 600nm in the spectrophotometer (Molecular Devices), being that OD_600_ = 1 corresponds to 8x10^8^ bacteria/ml.

### Bacterial Challenging and Colony-Forming Units (CFU) Assay

Mice received *E. coli* GFP^+^ 5x10^5^ bacteria/20g intravenously in 50µl. After the E. coli challenge, the control and 24 hours group survival rates were evaluated by monitoring mice every 3 hours for 30 hours. For CFU assay, 24 hours after the challenging mice were anesthetized, blood and liver were harvested, processed, and cultivated for 24h in Luria-Bertani agar (Sigma-Aldrich) containing ampicillin at 37°C. Next, CFUs were quantified, adjusting to blood volume (ml) and liver weight (g).

### Blood Clearance Assay

To evaluate the bacterial removal from blood, mice received intravenously *E. coli* GFP^+^ 5x10^6^ bacteria/20g in 50µl. After 5 min, mice were anesthetized, blood was collected from the hepatic vein diluted in PBS, and bacteria count was analyzed using Accuri™ C6 (BD Biosciences). The result was represented as the number of events per blood volume (µl).

### *Escherichia Coli* Tracking in the Liver

The real-time behavior and displacement of *E. coli* GFP^+^ were performed by tracking the bacteria in the liver using Intravital Microscopy. See the specific section for IVM. Briefly, mice placed in the microscope received 5x10^7^ bacteria/20g in 50µl after film acquisition started. Bacteria reach the liver in the first 20 seconds, and individualized events were followed for 5 minutes. The tracking of each *E. coli* GFP^+^ was performed by the NIS-Elements Advanced Research software using the NIS.ai module (Nikon). The circular graph contains an internal circle of 400 µm and an outer circle of 800 µm.

### Pharmacological Depletion of Kupffer Cells

Depletion of Kupffer cells was performed by intravenous injection of 50 µl, 125 µl, or 200 µl of Clodronate Liposomes (50mg/ml, Liposoma, Amsterdam, NL) (36) to achieve a depletion grade. The Control group received 0.9% saline solution in the same volumes. Flow Cytometry and Intravital Microscopy were used to accomplish the depletion. Mice were also submitted to blood clearance assay.

### Intravital Microscopy

Intravital microscopy (IVM) of the liver is powerful in seeing the biological phenomena *in vivo* and in real-time (37). Here, the Nikon Ti A1R confocal microscope (Nikon) was used. Before the surgical procedure, mice, when specified, received anti-F4/80 (clone T45-2342, BD Biosciences), anti-CD31 (clone 390, Biolegend) intravenously, and waited 20 minutes. Then, when discriminated, immediately before the anesthesia, mice received intravenously Sytox green (250nmol/kg, Invitrogen, Waltham, MA, USA). Next, mice were anesthetized as previously described (Liver non-parenchymal cells isolation section), followed by a midline laparoscopy to expose the liver for imaging. Mice were placed over an acrylic plate and then adequately positioned in the microscope. Finally, the positive staining was confirmed by injecting proper isotypes controls (IgG) for each staining. Movies and images were acquired using the NIS-Elements Advanced Research software using the NIS.ai module (Nikon).

### Immunofluorescence

Liver fragments were collected from the Control, 12, 24, and 72 hours groups and first, fixed overnight in PFA 4%, dehydrated in a sucrose gradient (10%-30%, weight/volume in PBS), and then embedded in Tissue-Tek ^®^ O.C.T compound (Sakura, Torrance, CA, USA). Cryosections of 15μm thickness were generated for each sample. For immunostaining, cryosections were permeabilized with 0.5% Triton X-100/PBS for 15 min and then incubated for 1 hour in the blocking solution (PBS containing 5% goat serum and 5 μg/ml mouse BD Fc Block). Targets were labeled overnight at 4°C using rat anti-F4/80 (clone T45-2342, BD Biosciences), rabbit anti-cleaved caspase 3 (clone 5A1, Cell Signaling Technology, Danvers, MA, USA), rabbit anti-Ki67 (Abcam, Cambridge, UK) primary antibodies, diluted 1:100 in PBS containing 0.05% Triton X-100. After washing in PBS, sections were incubated for 90 min at room temperature with the Alexa Fluor 488 conjugated goat anti-rabbit antibody (polyclonal, Invitrogen) at 1:200 dilution in PBS. Cell nuclei were stained with 4′,6-diamidino-2-phenylindole (DAPI, Sigma-Aldrich). Images were acquired using the Nikon Ti A1R confocal microscope (Nikon) and the NIS-Elements Advanced Research software (Nikon).

### Bone Marrow-Derived Macrophages Culture and Activation

Bone marrow precursors were harvested from femurs of LysM-TdTomato and wild-type mice, washed and differentiated into macrophages in a culture dish containing DMEM-F12 (Gibco) supplemented with 10% FBS (Gibco), 2mM of L-glutamine (Gibco), 100 µg/ml streptomycin (Gibco), 100 U/l penicillin (Gibco), 25mM HEPES (Sigma-Aldrich), and 20% of supernatant from L929 cell culture as previously described (38). After 7 days of differentiation at 37°C, 5% CO_2_ incubator, cells were harvested and stimulated overnight with IL-4 (20ng/ml, Peprotech, Rocky Hill, NJ, USA) to achieve the alternative activation (BMDM A.A). The activation status was analyzed by mRNA expression using real-time PCR.

### Analysis of mRNA Expression by Real-Time PCR

Total mRNA was extracted from BMDM, steady-state (BMDM ϕ) and alternatively activated (BMDM A.A) after overnight incubation using the ReliaPrep RNA Miniprep System (Promega, Madison, WI, USA) following the kit instructions. Then, total mRNA was quantified using a Nanodrop Lite (Thermo Fisher, Waltham, MA, USA) followed by reverse transcription using the iScript cDNA Synthesis kit (Bio-rad, Hercules, CA, USA) in a Rotor-Gene Q (Qiagen, Hilden, Germany) using manufacturer’s instructions. The amplification was performed using the Rotor-Gene Q (Qiagen), and the results were analyzed by the comparative threshold cycle method using 2 ^-ΔΔCT^ to determine the fold increase. Evaluated genes and their sequences are described in **Table 1**. Each gene expression was normalized to *actinb* (β-actin) mRNA expression as endogenous control and non-stimulated cells (BMDM ϕ).

**Table 1:** Primers sequence.

Gene	Foward	Reverse
*actinb*	5’-AGGTGTGCACCTTTTATTGGTCTCAA-3’	5’-TGTATGAAGGTTTGGTCTCCCT-3’
*chil3*	5’-AGAAGGGAGTTCAAACCTGGT-3’	5’-GTCTTGCTCATGTGTGTAAGTGA-3’
*rentla*	5’-AATCCAGCTAACTATCCCTCCA -3’	5’-CAGTAGCAGRCATCCCAGCA-3’
*arg1*	5’-CTGGCAGTTGGAAGCATCTCT-3’	5’-CTGGCAGTTGGAAGCATCTCT-3’

### Cell Therapy

Mice in the Acute Liver Injury model received the BMDM cell therapy intravenously 16 hours after APAP overdose. Cell suspension, 1x10^6^ cells in 100 µl, were administrated by orbital plexus that allows the rapid delivery to the liver (Data not shown, patronized by fluorescent beads administration). PBS was used as a vehicle, and the Control group received its volume. Flow Cytometry confirmed the cell delivery. Finally, control, 24 hours, BMDM ϕ, and BMDM A.A were analyzed at 24 hours after lesion induction (8 hours after treatment) by Flow Cytometry, Liver Histology, Intravital Microscopy, and Blood clearance assay.

### Data and Statistical Analysis

Biochemical assays were analyzed using SoftMax v5.4.1 (Molecular Devices). The NIS-Elements Advanced Research software was used to analyze and export all image, movie, and 3D data. The videos were generated using Sony Vegas Pro 16 (Sony, Tokyo, Japan). The Flow cytometry data were analyzed using FlowJo v10 (BD Biosciences). Data were analyzed using GraphPad Prism 8.0 (GraphPad Software, San Diego, CA, USA) and submitted for normality testing. The parametric data were shown as mean ± SEM and non-parametric as Median ± SD. In two-group comparisons, t-student or Mann-Whitney tests were performed. For kinetic or multiple groups assays, we performed One-way Anova followed by Tukey post-test in parametric data and Kruskal-Wallis followed by Dunn’s multiple comparison test for non-parametric data. Differences were considered statistically significant when p < 0.05.

## Results

### APAP Overdose Induces Liver Damage and Alteration in Myeloid Cell Composition

**Figure 1:**
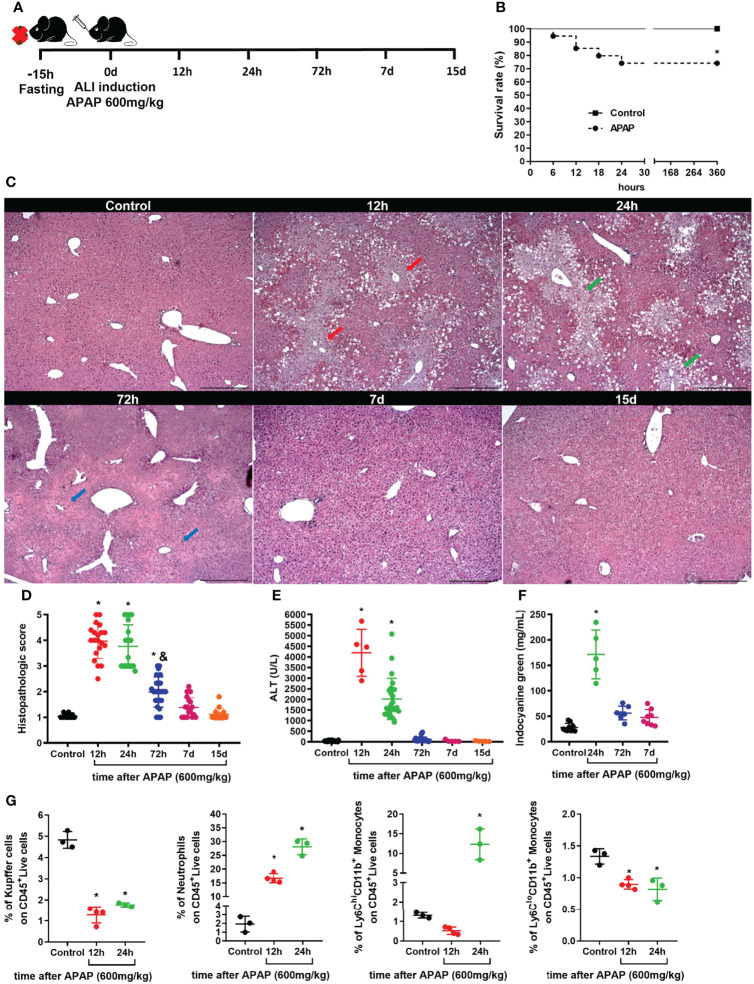
Acute liver injury induced by APAP overdose induces tissue damage and myeloid cell alterations. **(A)** Scheme for Acute Liver Injury (ALI) induced by Acetaminophen (APAP; 600mg/kg) overdose on 8-10 weeks old C57BL/6J mice. **(B)** Mice survival rate (%) after ALI induction; n ≥ 9 mice per group. **(C)** Representative liver histology of ALI kinetics stained with Hematoxylin and Eosin (H&E); scale bar = 500 µm. **(D)** Time-course of ALI tissue damage measured by the histopathologic score of liver sections; n = 8-15 mice per group. **(E)** Measurement of liver injury by serum levels of alanine aminotransferase (ALT); n = 5-15 mice per group. **(F)** Liver dysfunction showed by indocyanine green depuration from the serum; n = 5-9 mice per group. **(G)** Percentage (%) of different populations of myeloid cells within the CD45^+^ live cell pool during acute liver inflammation obtained by flow cytometry; n = 3-5 mice per group; Representative of 3 independent experiments. Data are presented as mean ± SEM. * indicates statistical difference to the control group using Mantel-Cox survival test **(B)** and one-way ANOVA followed and Tukey post-test **(D-G)** (* = p < 0.05). & indicates statistical difference compared to the 24h group using one-way ANOVA followed and Tukey post-test (& = p < 0.05).

Acetaminophen (APAP) is one of the most common causes of human acute liver injury and acute liver failure that can result in death (24). Here, we used a well-established mouse model of acute liver injury (ALI) (16, 33, 39) to investigate alterations in the myeloid cell compartment during liver damage. To induce ALI, C57BL/6 female mice were fasted for 15h and given oral gavage with 600 mg/kg of APAP diluted in warm saline **(** **Figure 1A** **).** In this model, APAP led to a 25% mortality in the first 24h **(** **Figure 1B** **)**, a similar mortality rate (28%) found in human acute liver failure induced by APAP overdose (24). Liver histopathology showed extensive necrotic areas during the acute phase, between 12h and 24h, followed by significant tissue regeneration after 72h of APAP administration, as indicated by the arrows in the respective histological sections of the liver (**Figure 1C** **)** and histopathologic score **(** **Figure 1D** **)**. According to liver histopathology, mice showed increased serum alanine aminotransferase (ALT) levels in the acute phase, which returned to baseline levels 72h after APAP administration and remained undetectable thereafter **(** **Figure 1E** **)**. The tissue damage caused by APAP overdose led to an impairment in liver function measured by an increase in indocyanine green (ICG) in the serum due to a reduced depuration rate 24h after APAP administration **(** **Figure 1F** **)**. Following 72h, the serum ICG levels were reduced to similar levels found in the non-treated control group **(** **Figure 1F** **)** in line with the regenerative process of the disease (24, 39, 40). To get an insight into the impact of the inflammatory response in the myeloid cell compartment, we assessed the influx of neutrophils in the liver and changes in the myeloid cell populations, including liver resident Kupffer cells (KCs), monocyte subsets **(** **Supplementary Figure 1A** **)**. We found that, during the acute phase of APAP overdose, the liver underwent a massive reduction of KCs, which was associated with a pronounced liver neutrophil infiltration and a significant increase in the Ly6C^hi^ monocytes and a slight decrease in Ly6C^lo^ monocytes **(** **Figure 1G** and **Supplementary Figure 1B** **)**. Taken together, our mouse model of ALI induced by APAP overdose was consistent with the human ALI disease outcome, showing induction of tissue damage and liver dysfunction associated with neutrophilic inflammation and substantial changes in liver myeloid cell composition.

### Reduction in Kupffer Cell Population During ALI Disrupts an Essential Niche in the Liver

**Figure 2:**
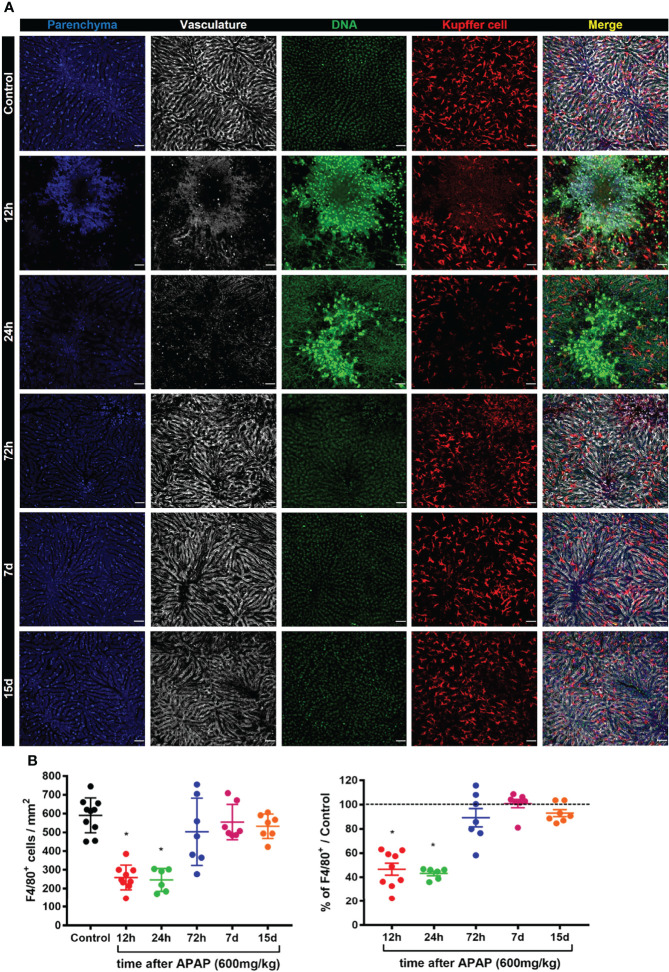
ALI causes a dramatic reduction in the Kupffer cell population and disrupts an essential niche in the liver. **(A)** Representative intravital microscopy (IVM) images acquired during ALI to characterize liver architecture and Kupffer cell population; Parenchyma is represented by liver auto-fluorescence (blue); Vasculature is stained by anti-CD31 PE antibody (white); Kupffer cell is stained by anti-F4/80 APC (red); DNA is stained Sytox green (green). Representative of 2 independent experiments; scale bar = 50 µm. **(B)** Kupffer cell quantification (Left panel) and the percentage based on Control (Right panel; —— = 100%); n ≥ 5 mice per group and 10 images per mice. Data are presented as mean ± SD. * indicates statistical difference compared to the control group using one-way ANOVA followed and Tukey post-test (* = p < 0.05).

Kupffer cells represent the biggest tissue-resident macrophage niche in adult mammals (5). These cells play a diverse and essential role in the liver, including iron metabolism and removal of gut-derived pathogens (2). Given the essential role of KCs in liver physiology and function, we characterized their phenotype, morphology, and positioning in liver-specific niche during the induction, progression and regeneration of the liver damage induced by APAP overdose. To visualize the alterations in the KCs morphology in a liver-specific niche during ALI, we used intravital microscopy of the liver in mice previously injected i.v. with fluorescent monoclonal antibodies (mAb) targeting the macrophage surface marker F4/80, as well as necrotic cells (DNA labeling by Sytox green) and liver vasculature using anti-CD31. **(** **Supplementary Figure 2A** **)**. Unbiased quantification of the KC morphology did not find a significant difference in KCs area **(** **Supplementary Figure 2B** **)**, length **(** **Supplementary Figure 2C**), elongation (**Supplementary Figure 2D**), and circularity (**Supplementary Figure 2D**) during the course of ALI. At steady-state, the liver parenchyma (blue, autofluorescence to 405nm laser) and the vasculature (white) was healthy with F4/80^+^ KCs (red) located inside the vasculature with projections to parenchyma **(** **Figure 2A** and **Supplementary Figure 3** **)**. In contrast, ALI was characterized by necrosis of the parenchyma after 12h and 24h of APAP overdose characterized by marked deposition of extracellular DNA in the necrotic areas **(** **Figure 2A** **)**. Moreover, both the liver vasculature structure and KCs were dramatically disrupted, especially in the necrotic areas **(** **Figure 2A** **)**. Seventy-two hours after APAP administration, the extracellular DNA deposition was completely cleared while the numbers of KCs and the liver parenchyma and vasculature structure started to show a process of restoration when compared to the Control group **(** **Figures 2A, B** **)**. On days 7 and 15 after ALI induction, all parameters resembled the Control group **(** **Figures 2A, B** **)**. These data demonstrate that APAP overdose causes a transient disruption in specific liver niches with a reduction in the number of KC during ALI, which can lead to increased susceptibility to infections.

**Figure 3:**
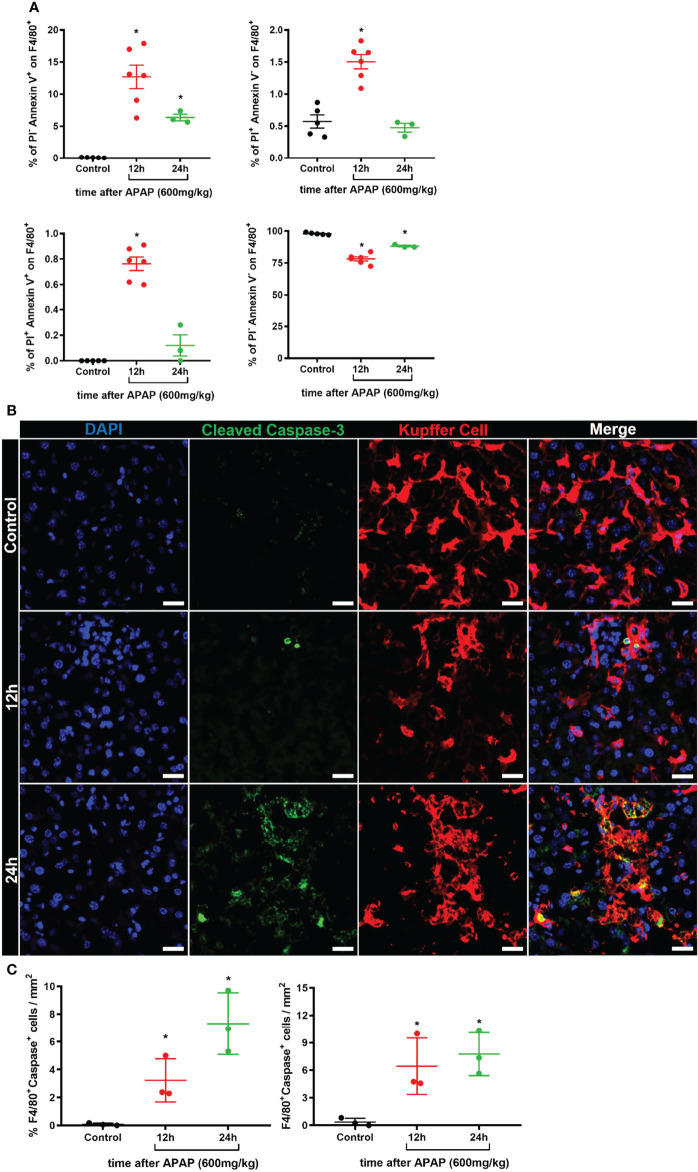
Kupffer cell disappearance occurs by induction of an apoptotic and cell death program during ALI. **(A)** Percentage of different Kupffer cell status of cell death: apoptosis (Upper left, Annexin V^+^PI^-^), necrosis (Upper right, Annexin V^-^PI^+^), necroptosis (Bottom left, Annexin V^+^PI^+^), and live cells (Bottom left, Annexin V^-^PI^-^); n ≥ 3 mice per group; Representative of 2 independent experiments. **(B)** Representative immunofluorescence images of liver sections stained for DAPI (Blue), Cleaved Caspase 3 (Green), and Kupffer cell (Red, anti-F4/80); scale bar = 20µm. **(C)** Quantification of the percentage (Left panel) and number (Right panel) of F4/80^+^Caspase-3^+^ Kupffer cells in liver sections; n ≥ 3 mice per group and 10 images per mice. Data are presented as mean ± SEM. * indicates statistical difference compared to the control group using one-way ANOVA followed and Tukey post-test (* = p < 0.05).

To better understand the biology of the KC disappearance in liver-specific niches during ALI, we investigate the cell death program of KCs. We first assessed whether KCs underwent apoptosis and cell death by flow cytometry using Annexin V and Propidium iodide (PI) staining of F4/80^+^ cells in the liver **(** **Supplementary Figure 4A** **)**. We found an increase in the percentage of apoptotic cells (Annexin V^+^PI^-^) 12h and 24h after APAP overdose and in the percentage of necrotic (Annexin V^-^PI^+^) and necroptotic cells (Annexin V^+^PI^+^) at 12h. **(** **Figure 3A** **)**. As expected, there was a reduction of live (Annexin V^-^PI^-^) F4/80^+^ cells at 12h and 24h of ALI **(** **Figure 3A** **)**. Furthermore, immunofluorescence of the liver for cleaved Caspase-3 (green), an intracellular cascade activated in cells undergoing apoptosis (41), revealed the activation of the apoptotic program in F4/80^+^ KC (red) in the first 12h and 24h of ALI **(** **Figure 3B** **)** and a significant increase in Caspase-3^+^ KC in the liver compared to the Control group **(** **Figure 3C** **)**. Therefore, Kupffer cell disappearance observed in ALI following APAP overdose occurs through an induction of an apoptotic and cell death program.

### A dual-input of cells occupies the liver macrophage niche during the resolutive phase

**Figure 4:**
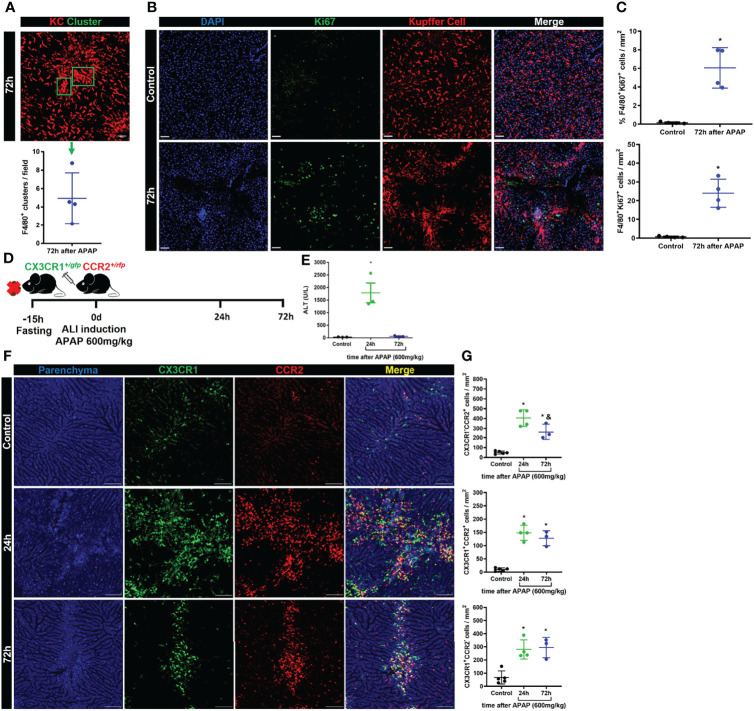
Kupffer cell proliferation and infiltration of monocyte-derived cells promote liver niche restoration. **(A)** Representative IVM image illustrating the Kupffer cell clusters and their quantification per field in the resolution phase, 72h after APAP administration; scale bar = 50 µm; n = 4 mice per group and 10 images per mice; representative of 2 independent experiments. **(B)** Evaluation of Kupffer cell proliferation during ALI resolution phase by immunofluorescence stained for DAPI (Blue), Ki67 (green), and F4/80 (Red); Representative images of 2 independent experiments; scale bar = 50 µm. **(C)** Quantification of % (Upper panel) and numbers (bottom panel) of Kupffer cell/Cluster co-localized with Ki67 staining; n = 4 mice per group and 10 images per mice; representative of 2 independent experiments. **(D)** Scheme of ALI induction by oral gavage administration of APAP 600mg/kg in 8–10-weeks old CX3CR1^/+GFP^+^^CCR2^/+rfp^ female mice. **(E)** Measurement of liver injury by serum levels of Alanine aminotransferase (ALT); n =3 mice per group; representative data for 2 experiments. **(F)** Representative images of IVM assay during ALI to characterize liver architecture (Parenchyma) and monocyte populations; Parenchyma is represented by liver auto-fluorescence (blue); monocytes CX3CR1^+^CCR2^+^ are represented by yellow cells; monocytes CX3CR1^+^CCR2^-^ are represented by green cells; monocytes CX3CR1^-^CCR2^+^ are represented by red cells; scale bar = 100 µm. **(G)** Quantification of each monocyte subset: monocytes CX3CR1^-^CCR2^+^ (Upper panel), monocytes CX3CR1^+^CCR2^+^ (Middle panel), and monocytes CX3CR1^+^CCR2^-^ (Bottom panel); n ≥ 3 mice per group and 10 images per mice; representative of 2 independent experiments. Data are presented as mean ± SEM. * indicates statistical difference to the control group using one-way ANOVA followed and Tukey post-test (* = p < 0.05). & Indicates statistical difference compared to the 24h group using one-way ANOVA followed and Tukey post-test (& = p < 0.05).

In homeostasis, liver macrophage niche maintenance occurs by self-renewal of the KCs with minimal input of monocyte-derived macrophages (11). However, monocyte-derived macrophages can contribute to niche recovery after a reduction in the KCs population (9). In **Figure 2** we showed that the KC niche starts to recover 72h after APAP overdose. During intravital microscopy imaging of the liver, we often observed the presence of cell clusters being formed in the resolutive stage, 72h after APAP overdose, with significant amounts of KC clusters (F4/80^+^ cells) per field of view **(** **Figure 4A** **)**. Therefore, KCs self-renewal was initially analyzed to address its contribution to the niche recovery. To do so, KCs proliferation was assessed by analysis of the intracellular marker of active cell proliferation Ki67 by immunofluorescence of the liver. Replicating Ki67^+^ KCs were detected 72h after APAP overdose with a significant increase over the Control **(** **Figures 4B, C** **)**, indicating a contribution of self-renewing Kupffer cells to niche recovery. Next, we looked at the monocyte infiltration and their positioning in the liver during ALI, as they could contribute to niche replacement as monocyte-derived macrophages. As previously described, there is a phenotypic and functional transition of different subtypes of monocytes until they reach the Kupffer cell niche and differentiate into monocyte-derived macrophages after ALI (20) or myeloid cell depletion (9). To investigate the monocytic transition and location during ALI, we used dual reporter CX3CR1^+/GFP^+^^CCR2^+/rfp^ mice in our mouse model of ALI induced by the APAP overdose **(** **Figure 4D** **)**. As expected, APAP administration led to an increased serum ALT levels, a sign of liver injury, at 24h, which returns to basal levels at 72h post-APAP overdose **(** **Figure 4E** **)**. Intravital microscopy analysis of CX3CR1^+/GFP^+^^CCR2^+/rfp^ mice also showed considerable parenchyma damage (blue) indicating necrotic areas after 24h of APAP overdose **(** **Figure 4F**), similar to our findings in wild-type mice in **Figure 2**. More interestingly, there was an extensive infiltration of inflammatory monocytes (CX3CR1^-^CCR2^+^) and patrolling monocytes (CX3CR1^+^CCR2^-^) and fewer monocytes with a transitory phenotype (CX3CR1^+^CCR2^+^) in the liver after 24h **(** **Figures 4F, G** **)**. Seventy-two hours after APAP overdose, we observed a significant reduction in the inflammatory monocyte population compared to the acute phase, at 24h, while the transitory and patrolling monocyte populations were unaltered **(** **Figures 4F, G** **)**. It is worth highlighting that these different monocyte populations found in the acute and resolution phase were positioned in the necrotic areas represented by disrupted parenchyma **(** **Figure 4F** **)**. Moreover, we did not find KCs within the necrotic areas. Therefore, in addition to the proliferation of remaining KC (Ki67^+^ KC), monocytes strategically located in damaged areas can give rise to new macrophages during ALI, supporting the notion of a KCs/monocyte-derived macrophage dual-input in liver regeneration.

### Liver firewall function is impaired during ALI, increasing the susceptibility to bacterial infections

**Figure 5:**
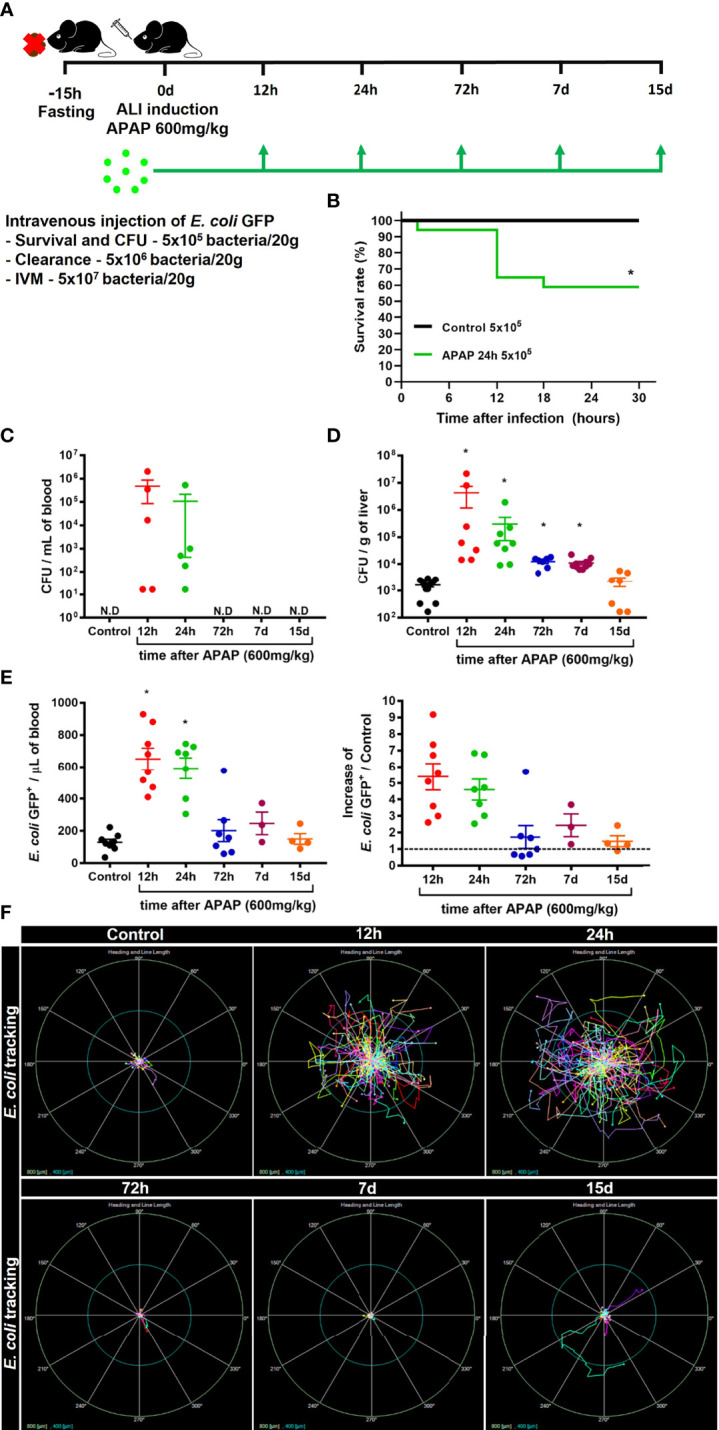
ALI impairs liver firewall function and promotes susceptibility to bacterial infection. **(A)** Scheme for intravenous (i.v) injection of *E. coli* bacteria during the time-course of ALI on 8-10 weeks old C57BL/6J mice. **(B)** Mice survival rate (%) after infection with 5x10^5^ bacteria/20g in APAP untreated control mice or in mice 24h after APAP overdose (APAP 24h group); n ≥ 5 mice per group. Quantification of Colony-forming units (CFU) of *E. coli* in the blood **(C)** and liver **(D)** 24h after infection with 5x10^5^ bacteria/20g; n ≥ 9 mice per group; Representative of 3 independent experiments. **(E)** Measurement of liver firewall function by quantification of GFP^+^ bacterial events from blood samples (Left panel) by Flow cytometry and determination of the blood clearance ability (Right panel; Increase over the control (-------- = 1) 5 minutes after infection with 5x10^6^ bacteria/20g; n ≥ 3 mice per group; Representative of 3 independent experiments. **(F)** In vivo real-time tracking of *E. coli* by Intravital Microscopy (IVM) during the first 5 minutes after infection with 5x10^7^ bacteria/20g; Representative bacterial displacement graphs for each group; scale = 800 µm; n ≥ 5 mice per group. Representative of 2 independent experiments. Data are presented as mean ± SEM. * indicates statistical difference compared to the control group using Mantel-Cox for survival test **(B)**, non-parametric Kruskal Wallis test followed by Dunn's multiple comparisons tests **(C, D)**, and one-way ANOVA followed and Tukey post-test **(E)** (* = p < 0.05).

Liver firewall function is one of the primary immunological functions of the liver, which consist of removing gut-derived compounds, exogenous molecules, or pathogens from the bloodstream (42). Indeed, it has been reported that patients diagnosed with ALI often develop systemic infection by bacteria or fungi (25), indicating an impairment of liver function. Thus, we hypothesized that disruption of the hepatic tissue and myeloid cell niche alteration could increase the susceptibility to infections. To test this hypothesis, mice were challenged with intravenous injection of *E. coli* expressing GFP^+^ during ALI **(** **Figure 5A** **)**. First, we measured the mortality rate in mice injected with a low dose of *E. coli* (5x10^5^ bacteria/20g) without or with APAP overdose. While 100% of mice without APAP administration survived after *E. coli* infection, we found a 40% mortality rate in mice treated with APAP, showing increased susceptibility to systemic infection in the APAP overdose group **(** **Figure 5B** **)**. Next, we addressed the ability of the liver to clear circulating *E. coli*. To do so, mice treated with APAP overdose were injected with 5x10^5^ *E. coli*/20g in different time points (12h, 24h, 72h, 7d, and 15d) and, 24h later, a CFU assay was performed. CFUs were only detected in blood samples from mice 12h and 24h after APAP overdose when liver injury peaks **(** **Figure 5C** **).** Accordingly, liver samples from 12h and 24h post-APAP overdose groups displayed the highest increase in CFU **(** **Figure 5D** **)**, indicating an inability to control the bacterial growth. Surprisingly, 72 hours and 7 days post-APAP overdose, when both liver ALT and histopathology were diminished or returned to baseline, we still detect enhanced CFUs in the liver **(** **Figure 5D**), suggesting that an extended period might be necessary for the hepatic immune system to return into homeostasis as observed when mice were infected at day 15.

Next, to evaluate how ALI affects the bacterial clearance from the blood by the liver, mice received an i.v injection of 5x10^6^ *E. coli* GFP^+^/20g, and 5 minutes later, the blood was collected from the hepatic vein, and then GFP^+^ events were counted by flow cytometry **(** **Supplementary Figure 5** **)**. In line with our previous results, we found an impairment in bacterial clearance from the blood during the acute phase (12h and 24h) of ALI, as showed by increased amounts of circulating GFP^++^ bacteria, approximately a five-fold increase over the control group **(** **Figure 5E** **)**. During the resolution phase, 72h post-APAP administration, we observed an efficient clearance of circulating bacteria by the liver, comparable to the uninfected control group **(** **Figure 5E** **)**. To evaluate the real-time behavior of circulating bacteria in the liver, we performed IVM to track the bacteria *in vivo* after i.v injection of 5x10^7^ *E. coli* GFP^+^/20g. Consistent with the impaired clearance capacity in the acute phase of ALI, *E. coli* real-time tracking using IVM assay revealed a higher bacterial displacement 12h and 24h after APAP-induced liver injury **(** **Figure 5F** **)**. On the other hand, tracking *E. coli* at later time points, starting at 72h post-APAP, showed limited bacterial displacement in the liver **(** **Figure 5F** **)**, indicating a rapid capture of bacteria by liver phagocytes. Thus, impaired blood clearance of pathogens by the liver during acute stages of ALI suggests a susceptibility window for systemic infections during liver damage induced by APAP overdose.

### Kupffer Cells are Essential for the Liver Firewall Function, Which is Reduced During Acute Liver Injury

**Figure 6:**
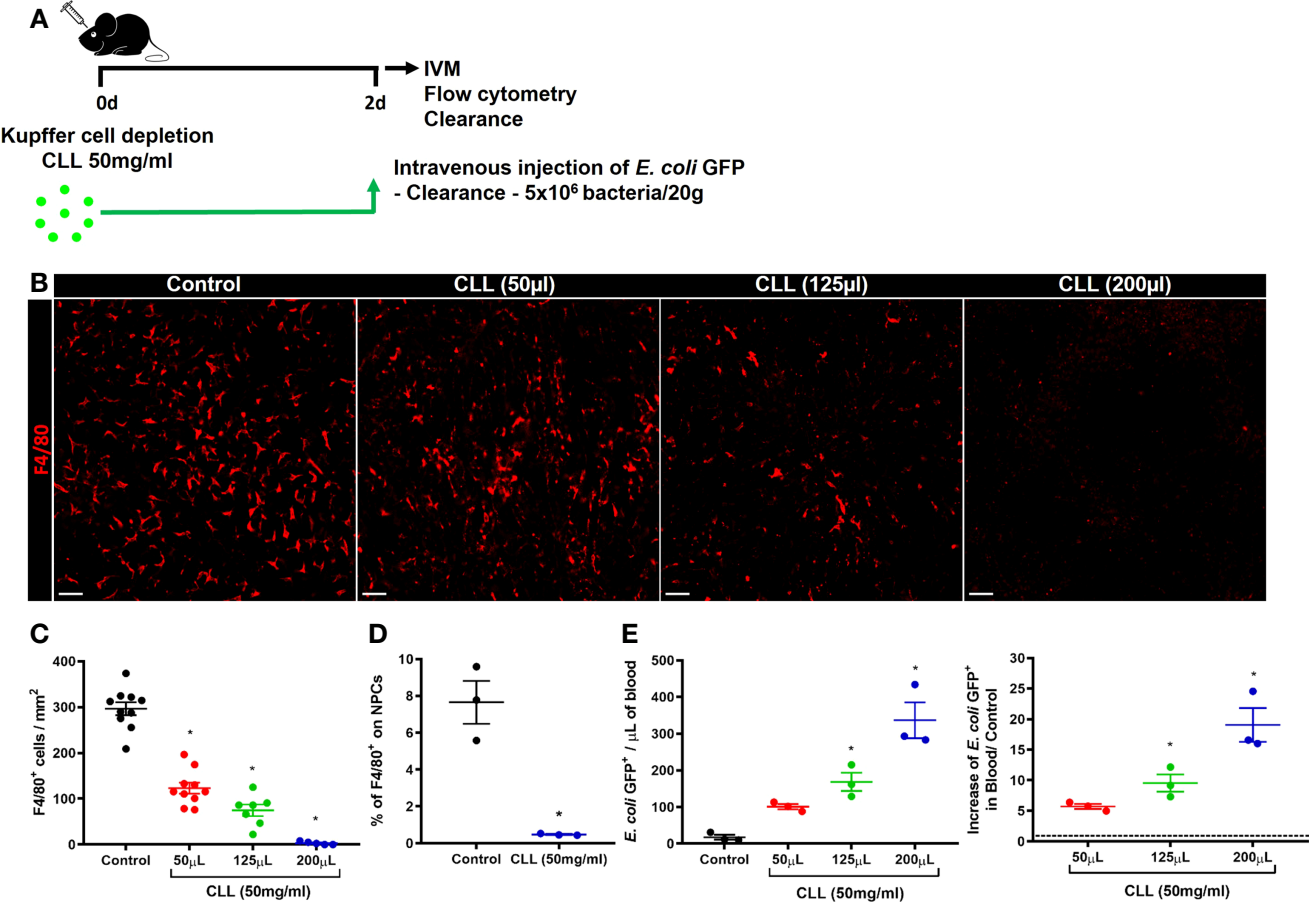
Kupffer cells are essential for bacterial clearance and liver firewall function. **(A)** Scheme for Kupffer cell depletion using i.v injection of Clodronate Liposomes (CCL; 50mg/ml) in different volumes (50 µL, 125µL, and 200 µL). **(B)** Representative IVM images in mice intravenously injected with anti-F4/80 (Kupffer cells in red) 20 minutes before surgery; scale bar = 100 µm. **(C)** Quantification of Kupffer cells per mm^2^; n ≥ 5 mice per group and 10 images per mice. **(D)** Percentage of F4/80^+^ cells in liver non-parenchymal cells (NPCs) measured by Flow Cytometry. n = 3 mice per group; Representative of 2 independent experiments. **(E)** Quantification of *E. coli* GFP^+^ events from blood samples (left panel) by flow cytometry and determination of the blood clearance ability (right panel); n ≥ 3 mice per group; Representative of 2 independent experiments. Data are presented as mean ± SEM. * indicates statistical difference compared to the control group using T-Student test **(D)** and one-way ANOVA followed and Tukey post-test **(C**, **E)** (* = p < 0.05).

Phagocytes are critical for the capture and elimination of pathogens. In the liver, KCs are the primary phagocyte responsible for those functions (42). To test whether the impairment in bacterial clearance during the acute stages of ALI was due to the reduced number of KCs, we pharmacologically depleted liver phagocytes by using intravenous injection of Clodronate Liposomes (CLL) **(** **Figure 6A** **)**. Although *in vivo* CLL treatment can deplete phagocytes other than KCs, this method is still reliable for studies on Kupffer cell biology (34). IVM and flow cytometry analyses showed a dose-dependent depletion of KCs in the liver 48h after treatment **(** **Figures 6B–D**). The reduced number of KCs in the liver was inversely related to the increase in bacterial load in the blood, as showed by the increase in *E. coli* GFP^+^ events in blood samples measured by flow cytometry **(** **Figure 6E** **)**. These data demonstrate a critical role of liver phagocytes, in which KCs are the most abundant, in blood bacterial clearance and liver firewall function.

**Figure 7:**
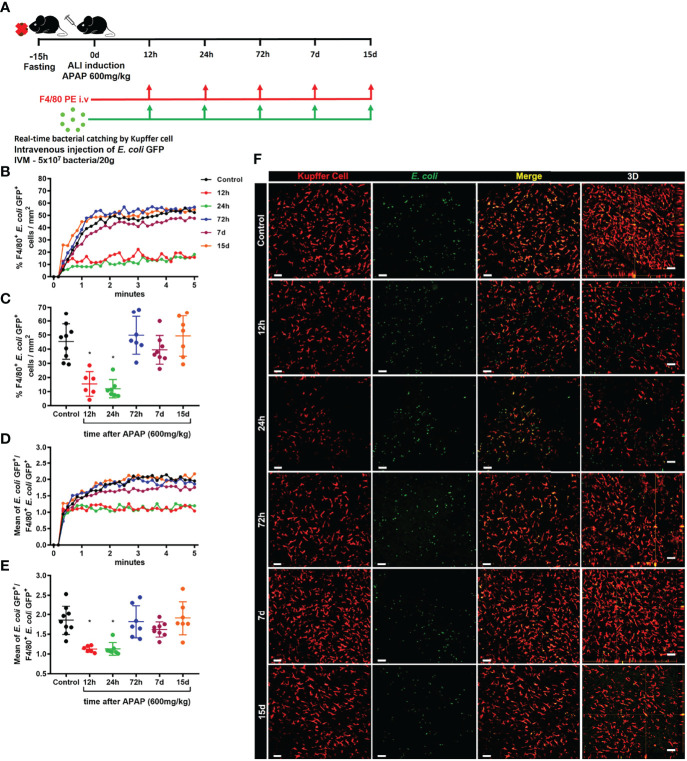
Kupffer cell phagocytic ability is strongly reduced in the early stage of ALI. **(A)** Scheme of real-time *E. coli* phagocytosis by Kupffer cells in 5 minutes after i.v injection of 5x10^7^ bacteria/20g. Measurement of real-time *E. coli* phagocytosis by Kupffer cell every 10 seconds during the first 5 minutes **(B)** after injection and at the end of 5 minutes **(C)**; n ≥ 6 mice per group; representative of 2 independent experiments. Mean of *E. coli* GFP^+^ numbers per Kupffer cell GFP^+^ quantified during the first 5 minutes **(D)** after injection and at the end of 5 minutes **(E)**; n ≥ 6 mice per group; representative of 2 independent experiments. **(F)** Representative images at minute 5 and 3D slice (fourth column) for each group; *E. coli* is green; Kupffer cell is stained by anti-F4/80 PE (red) and *E. coli* merged with Kupffer cell in yellow; scale bar = 50 µm. Data are presented as mean ± SD. * indicates statistical difference compared to the control group using one-way ANOVA followed and Tukey post-test (* = p < 0.05).

We next evaluated the phagocytosis capacity of KCs during ALI. For this purpose, *in vivo* real-time catching of intravenously injected *E. coli* GFP^+^ by KCs during the time-course of ALI was assessed by IVM **(** **Figure 7A** **)**. During homeostasis, KCs rapidly phagocytosed circulating bacteria. This capacity is visible reduced 12h and 24h after APAP-induced lesion, but begins to be restored after 72h and normalized 7 and 15 days after APAP overdose **(** **Supplementary Video 1**). With this approach, we confirmed that KCs play a vital role in the clearance of circulating pathogens, such as *E. coli*. We also quantified the percentage of KCs that captured circulating *E. coli* GFP^+^ during the acute phase of ALI. We found that the percentage of KCs that phagocytosed *E. coli* GFP^+^ (F4/80^+^ *E. coli* GFP^++^ cells) was significatively reduced in the first 12h and 24h of APAP overdose, which returned to a similar percentage after 72h **(** **Figures 7B, C** **)**. In addition, KCs from mice 12h and 24h after APAP overdose internalize fewer circulating bacteria, showed as a mean of *E. coli* GFP^++^ per F4/80^+^ *E. coli* GFP^++^ cells. The number of internalized bacteria by KCs from mice at 72h, 7d and 15d after APA-induced ALI was comparable to control mice **(** **Figures 7D, E** and **Supplementary Video 2** **)**. Altogether, these data showed that KCs play a critical role in bacterial clearance from the blood due to their high phagocytic capacity and suggest that disruption of the Kupffer cell niche by APAP overdose is an important cause of increased susceptibility to infections during ALI.

### Bone Marrow-Derived Macrophage Therapy Reduces Liver Necrosis and Improves the Liver Firewall Function During ALI

**Figure 8:**
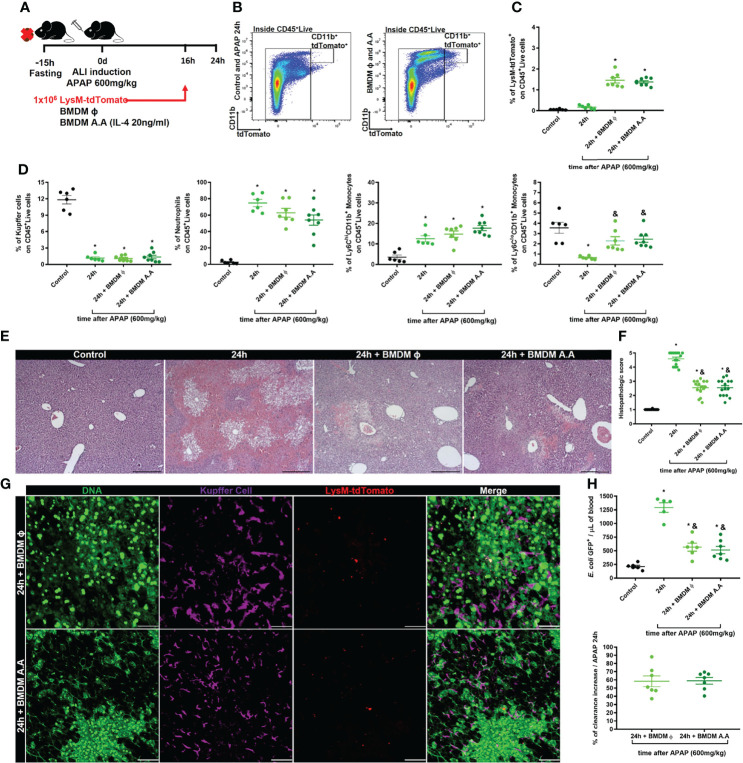
Cellular therapy with bone marrow-derived macrophages reduce liver necrosis and rescues liver blood clearance. **(A)** Scheme for bone marrow-derived macrophage (BMDM) therapy during ALI, 16h after APAP administration. 1x10^6^ BMDC from LysM^cre/+^Rosa26^tdTomato/+^ mice that were non-activated (BMDM ϕ) or alternatively activated with IL-4 (BMDM A.A) were transferred into recipient mice 16h after APAP administration. **(B)** Flow cytometry gate strategy of inoculated LysM^cre/+^Rosa26^tdTomato/+^ BMDM ϕ and BMDM A.A in CD45+ live cell gate detected in the liver 8h after BMDM therapy (24h post-APAP administration). **(C)** Percentage of LysM^cre/+^Rosa26^tdTomato/+^ in liver samples; n ≥ 5 mice; representative of 2 independent experiments. **(D)** Evaluation of myeloid cell niche after BMDM cell therapy by flow cytometry showing the percentage (%) of myeloid cell subtypes; n ≥ 5 mice; representative of 2 independent experiments. **(E)** Representative liver histopathology of all four groups stained with Hematoxylin and Eosin (H&E); scale bar = 500 µm. **(F)** Tissue damage measured by the histopathologic score of the liver; n = 8-15 mice per group. **(G)** Representative IVM images of treated groups (BMDM A.A and BMDM ϕ) showing extracellular DNA (green, Sytox green), Kupffer cell (purple, anti-F4/80), and LysM^cre/+^Rosa26^tdTomato/+^ (red); scale bar = 100 µm; n = 2 mice per group. **(H)** Measurement of liver firewall function by quantification of *E. coli* GFP^+^ events per ul of blood (Upper panel) by Flow cytometry and determination of the blood clearance ability (Bottom panel; % increase over the APAP at 24h) 5 minutes after infection with 5x10^6^ bacteria/20g; n ≥ 5 mice per group; Representative of 2 independent experiments. Data are presented as mean ± SEM. * indicates statistical difference compared to the control group using one-way ANOVA followed and Tukey post-test (* = p < 0.05). & indicates statistical difference compared to the 24h group (not treated with BMDM) using one-way ANOVA followed and Tukey post-test (& = p < 0.05).

Liver macrophage niche replacement and organ recovery by cell therapy with bone marrow-derived macrophages have been proposed as a promising treatment for liver fibrosis and APAP overdose (31, 32). We, therefore, hypothesized that the liver macrophage niche recovery or replacement during the first 24h of ALI could reduce the susceptibility to infection from circulating pathogens. Thus, we treated mice intravenously with 1x10^6^ bone marrow-derived macrophages at steady-state (BMDM ϕ) or after alternative activation with IL-4 (BMDM A.A) 16h after APAP-induced liver injury. The effect of the therapy with bone marrow-derived macrophages in liver recovery was analyzed during the peak of ALI, 24h after APAP overdose administration **(** **Figure 8A** **)**. First, we assessed the BMDM activation status by real-time PCR. We found that IL-4 by itself was enough to induce an alternative activation profile in (BMDM A.A) as showed by increased mRNA expression of *chil3*, *retnla*, and *arg1* when compared to BMDM ϕ **(** **Supplementary Figure 6A** **)**. Next, we confirmed by flow cytometry that the transferred fluorescent-reporter BMDMs reached the target organ, the liver **(** **Figure 8B** **)**. We found that both BMDM ϕ and BMDM A.A equally arrived in the organ **(** **Figure 8C** and **Supplementary Figure 6B** **)**, and the BMDMs were localized in the liver 8h after cell transfer.

We also evaluated the myeloid cell compartment and the neutrophilic inflammation in the liver of APAP-treated mice by flow cytometry 8h after BMDM transfer. BMDM therapy did not alter the percentage and the absolute number of Kupffer cells, neutrophils, and inflammatory monocytes (Ly6C^hi^CD11b^+^ cells) compared to APAP-treated mice that did not receive BMDM transplantation **(** **Figure 8D** and **Supplementary Figure 6C** **)**. However, the BMDM therapy in APAP-treated mice did recover the percentage **(** **Figure 8D** **)** and number **(** **Supplementary** **Figure 6C** **)** of patrolling monocytes (Ly6C^lo^CD11b^+^ cells) in the liver to levels comparable to the APAP-untreated Control group. Patrolling monocytes are often characterized as having a pro-regenerative phenotype (43), acting in the resolution phase of ALI (20). Thus, we investigated whether the increased numbers of patrolling monocytes (Ly6C^lo^CD11b^+^ cells) reduced the liver damage caused by APAP overdose. Histopathologic analysis of the liver showed reduced necrotic areas around centrilobular veins in BMDM ϕ and BMDM A.A groups and those areas did not connect to adjacent necrotic areas, as was seen in mice that did not receive BMDM therapy **(** **Figure 8E** **)**. In addition, the therapy with both the steady-state and alternatively activated macrophages reduced the histopathologic score due to the reduction of necrotic areas and increased regeneration of the damaged organ compared to the non-treated group **(** **Figures 8E, F** **)**. Intriguingly, we did not observe a significant change in levels of ALT in the serum **(** **Supplementary Figure 6C** **)**, as has been previously observed by another group (32). We next evaluated the positioning of the transferred BMDMs in the liver during ALI using IVM. We found that BMDM ϕ and BMDM A.A were positioned in the necrotic areas, characterized by deposition of extracellular DNA, where the number of KCs were reduced **(** **Figure 8G** **)**, suggesting that BMDM ϕ and BMDM A.A occupied the niche opened by KCs death during ALI. Finally, we evaluated the liver firewall function using the *in vivo* assay of bacterial clearance from the blood. We found that therapy with both BMDM ϕ and BMDM A.A in mice intravenously infected with *E. coli* GFP^+^ reduced the number of bacteria in the blood compared to *E. coli* GFP^+^ infected mice without BMDM therapy **(** **Figure 8H**, upper panel), promoting an increase of 60% in liver blood clearance capacity **(** **Figure 8H**, bottom panel). Taken together, these data demonstrated that therapy with BMDMs, which occupy the open niche in the liver caused by KCs death after APAP overdose, improves the regeneration of the damaged liver during ALI, decreasing the susceptibility to systemic infections.

## Discussion

Acetaminophen overdose induces a complex acute inflammation initiated by hepatocyte necrosis, leading to massive parenchyma damage (16). Consequently, intracellular components are released within the liver and can reach the systemic circulation acting as DAMPS. In addition, since the liver is an extremely vascularized organ, hepatic microcirculation – together with other resident immune cells – is profoundly disturbed during ALI (19, 33). These events lead to inflammation that, if not controlled, can evolve into a fatal outcome (24). Patients that survived the acute metabolic damage due to liver necrosis but have subsequent systemic infections are responsible for a significant percentage of the mortality rate of those patients (25, 44). These conditions are mainly related to the loss of blood clearance by the liver, which is crucial for restraining the circulation of pathogens. A key liver component for such firewall function are KCs (3, 42), and it is becoming increasingly clear that disruption in their number or function are a hallmark of liver failure.

Liver necrosis results in an abrupt release of intracellular components, such as ALT, which are used for laboratory diagnosis of liver damage. However, several intracellular molecules, which comprise a large group of damage-associated molecular patterns (DAMPs), are also released (18). In fact, DNA, HMGB-1 (high mobility group box protein 1), and histones are not only responsible for acute inflammation, but also for direct endothelial cell death (45), damaging the hepatic sinusoidal network. Therefore, necrosis-derived DAMPs could consequently trigger KCs death since they are mainly located inside liver sinusoids. In sharp contrast, the disappearance of tissue-resident macrophages during local inflammation has been also discussed as “intentional” cell death, which could avoid over inflammation and further tissue damage (46, 47). The KCs death phenomenon also occurs in various liver inflammatory processes, such as viral or bacterial infection and sterile inflammation caused by non-alcoholic steatohepatitis (12, 14, 15). In ALI induced by APAP overdose, KC death occurs through an apoptotic program, which a marked characteristic of this program is the reduced amplification of local inflammation (48). However, in the absence of KCs, the ALI outcome is worse, indicating a crucial role of those cells in restraining the onset of APAP overdose-induced disease (49, 50). In addition, KCs also are known to participate in the resolution of the inflammation and to contribute to organ regeneration by removing debris and stimulating angiogenesis, respectively (51, 52). Thus, in the absence of functional KCs, tissue damage during ALI may worsen and organ regeneration may be delayed.

Phagocytosis and the elimination of pathogens are primary KC functions (3). Here, we highlighted the importance of this function during the ALI. Following APAP-induced liver injury, we observed a reduction in the KC population and a reduced percentage of cells in the liver with the ability to capture circulating bacteria. This defect in host liver-resident immune cell ability to clear blood-born bacteria remained impaired up to 7 days post-APAP overdose. Therefore, the loss of such vital liver function might open a window for systemic infections in patients with ALF induced by ALI (24). We have previously shown that mice treated with APAP had no detectable bacteria in blood and liver samples, suggesting that APAP-induced liver injury does not promote microbiota translocation by itself but rather would predispose patients to systemic infections following exposure to an infectious pathogen (Marques et al, 2015). Indeed, approximately 50% of ALF patients are affected by systemic infections, with a 40% of mortality rate (24, 25, 44). Thus, disruption of the KC niche appears to be a determinant factor for worse outcomes in patients with ALF.

During an acute inflammatory response in the liver, the KCs replenishment kinetics must be increased relative to the steady-state (6) as the rapid resolution of inflammation and the return of the organ to homeostasis are essential for the organism. KCs depletion caused by liver inflammation can create an environment for a proper liver resident macrophage replenishment (29, 53). However, the origin of these new cells depends on the inflammatory insult and extension of the tissue damage and inflammation. It has been proposed that KCs replicate in the resolution phase of ALI and contribute to niche replacement (20), although this does not exclude the possibility of a dual input of liver cell replenishment, one from replicating KCs and another from infiltrating monocytes. Moreover, the transcriptional profile transitioned from the Ly6C^hi^ monocytes to Ly6C^lo^ monocytes and then to KC at 72 hours, indicating a phenotype changing from first to the last. Here, we showed that replicating KCs that remained in the liver formed cell clusters in necrotic areas. We also found a massive infiltration of Ly6C^hi^ and Ly6C^lo^ monocytes early in ALI that lasted until the resolution phase and were strategically positioned in areas devoid of KCs. Differentiation of circulating monocytes into liver resident macrophages has been described for other liver disease models (9, 12, 28, 49). Another study described a possible competition by the open liver macrophage niche between the remaining KCs and the infiltrated monocytes (29). In our study we were not able to definitively determine whether the recovery in KCs population in the resolution phase of ALI was due to replication of remaining liver KCs, infiltration of monocytes and their differentiation into new KCs, or both. An approach that could address this is the fate-mapping model *via* Ms4a3, which allows the tracking of monocyte-derived cells (11). Therefore, further studies are still necessary to definitively determine the KC replication and monocyte differentiation rate in the liver macrophage niche replenishment during ALI.

Differentiation of monocytes into functionally competent KCs requires a period to adapt to the tissue environment, including the acquisition of specific transcriptional and phenotypic characteristics and their positioning in specific organ niches (9, 10, 29, 54, 55). Infiltrating monocytes are transiently immature compared to KCs with signaling pathways related to phagocytosis and elimination of pathogens, such as FcγR signaling, complement receptors and their downstream cascade, phagosome and lysosome, downregulated in both Ly6C^hi^ and Ly6C^lo^ monocyte subsets (20). Thus, even with the KC niche transiently occupied by infiltrating monocytes, there is an increased susceptibility to systemic infections due to their inability to appropriately deal with circulating bacteria. Even though professional phagocytes internalize pathogens, the latter disposes of mechanisms to evade the cellular immune response (56). Pathogens, including bacteria, commonly use three strategies: I) escape of phagosome mechanisms; II) inhibition of the fusion between phagosome and lysosome; III) capacity to resist inside the phagolysosome (57, 58). This could explain why, in our system, the *E. coli* titers were increased in the liver 7 days after APAP overdose. In addition, despite the presence of remaining KCs in the injured liver, their failure to adequately phagocytose and eliminate pathogens could be explained by increased demand for removal of cell debris due to liver necrosis (51, 59, 60). Therefore, the infiltration of monocytes with an immature phenotype added to insufficient response by remaining KCs may result in impaired antimicrobial response during ALI, which can contribute to the enhanced susceptibility to infections (61).

The difficulty of proper and early diagnosis of the ALI and ALF reduces their treatment. However, a cell-based therapeutic possibility recently emerged as an alternative for managing hepatic diseases. In the last decade, macrophage therapy has been shown effective anti-fibrotic and pro-regenerative results (30, 31). In fact, treatment of APAP overdose using bone marrow-derived macrophages, generated by CSF-1 stimuli into precursors in culture, significantly improved the recovery of the injured liver. Also, alternatively activated BMDM (BMDM A.A), an anti-inflammatory activation profile (62), accelerated the resolution of inflammation by clearance of the necrotic areas and stimulation of hepatocellular proliferation when analyzed 36h after APAP-induced tissue lesion (32). Indeed, BMDM A.A. is highly phagocytic and expresses a pro-regenerative profile, such as IL-10, TGF-β, YM1, and FIZZ1 (32). Here, we extended the BMDM therapy effects to the acute phase of ALI. We found that the engrafted BMDM migrated to the liver, reduced liver necrosis, and improved the blood bacterial clearance capacity by the liver. However, therapy with BMDM alternatively activated did not differ from the therapy with conventional BMDM, suggesting that BMDM A.A. may not be necessary for the acute phase as they are for the resolution stage. Importantly, although the BMDM therapy did not immediately replenish KCs during ALI, it can support and maintain some KC functions while the repopulation and maturation processes occur. It is important to note that macrophage maturation and education (mainly due to tissue factors) can be a long-term process (30-60 days in mice) and, in some cases, may not fully restore the original macrophage identity. Therefore, despite of some limitations, BMDM therapy appears as a promising treatment for ALI and ALF in during both early and later stages.

In summary, we found that the liver macrophage niche is essential for restraining ALI and that BMDM therapy improves the resolution of liver inflammation and damage, as well as reduces the susceptibility of the host to infections. Thus, therapies targeting the Kupffer cell niche can add to the current treatment of the ALI and ALF symptoms in order to enhance liver healing and improve the recovery of organ function. Such a therapeutic strategy that can be extended to other diseases with disruption of tissue-resident macrophage populations.

## Ethics Statement

The animal study was reviewed and approved by Comissão Ética de Utilização Animal (CEUA), Universidade Federal de Minas Gerais (UFMG), protocols number 377/2016 and 076/2020.

## Author Contributions

ML conceptualized the project, designed and performed the experiments, analyzed and interpreted data, and wrote the manuscript. BN designed and performed the experiments, interpreted data, and wrote the manuscript. MM performed the experiments, interpreted data, and wrote the manuscript. GCS designed and performed the experiments. RM performed the experiments. PM performed the experiments. AO designed and performed the experiments and interpreted data. LF reviewed and wrote the manuscript. RG designed and supervised the experiments and interpreted data. GM conceptualized the project, supervised the experiments, interpreted data, and wrote the manuscript.

## Funding

This study was financed by CNPq, CAPES, and FAPEMIG (Universal 2018, Rede Mineira de Imunobiolo´gicos) agencies.

## Acknowledgments

The authors are indebted to Dra. Cristina de Paula for exquisite lab managing work at the Center for Gastrointestinal Biology; members of Macrophage and Monocyte Biology Laboratory/UFMG; Professor Leda Vieira for opening the Laboratory of Gnotobiology and Immunology to space and equipment usage; Dr. Matheus Batista Carneiro for all scientific discussions.

## Conflict of Interest

ML is a CNPq fellow. MM was a CAPES fellow and is currently an EILF-EASL Sheila Sherlock Postdoctoral fellow. BN is a CNPq fellow. RM is a CAPES. GCS was a CAPES fellow and is currently FRQS postdoctoral fellowship. PM is a CNPq fellow. AO is a FAPEMIG and CNPq fellow. RG is a FAPEMIG fellow (RED-00313-16; REMETTEC-RED 00570-16). GM is currently a CNPq fellow.

The authors declare that the research was conducted without any commercial or financial relationships that could be construed as a potential conflict of interest.

## Publisher’s Note

All claims expressed in this article are solely those of the authors and do not necessarily represent those of their affiliated organizations, or those of the publisher, the editors and the reviewers. Any product that may be evaluated in this article, or claim that may be made by its manufacturer, is not guaranteed or endorsed by the publisher.
