# Corrigendum: Susceptibility to infections during acute liver injury depends on transient disruption of liver macrophage niche

**DOI:** 10.3389/fimmu.2022.1038067

**Published:** 2022-10-14

**Authors:** Mateus Eustáquio Lopes, Brenda Naemi Nakagaki, Matheus Silvério Mattos, Gabriel Henrique Campolina-Silva, Raquel de Oliveira Meira, Pierre Henrique de Menezes Paixão, André Gustavo Oliveira, Lucas D. Faustino, Ricardo Gonçalves, Gustavo Batista Menezes

**Affiliations:** ^1^ Center for Gastrointestinal Biology, Departamento de Morfologia, Instituto de Ciências Biológicas, Universidade Federal de Minas Gerais (UFMG), Belo Horizonte, Brazil; ^2^ Department of Biochemistry and Immunology, Instituto de Ciências Biológicas, Universidade Federal de Minas Gerais (UFMG), Belo Horizonte, Brazil; ^3^ Centre de Recherche du Centre Hospitalier Universitaire de Québec, Université Laval, Québec, QC, Canada; ^4^ Macrophage and Monocyte Biology Laboratory, Department of Pathology, Instituto de Ciências Biológicas, Universidade Federal de Minas Gerais (UFMG), Belo Horizonte, Brazil; ^5^ Department of Physiology and Biophysics, Instituto de Ciências Biológicas, Universidade Federal de Minas Gerais (UFMG), Belo Horizonte, Brazil; ^6^ Center for Immunology and Inflammatory Diseases, Division of Rheumatology, Allergy and Immunology, Massachusetts General Hospital, Harvard Medical School, Boston, MA, United States

**Keywords:** Kupffer cell, Macrophage niche, Acute liver injury, Inflammation, Cell therapy, Systemic infection

In the published article, there was an error in affiliations 1, 2, 4 and 5. Instead of “Center for Gastrointestinal Biology, Department of Morphology, Institute of Biological Sciences, Federal University of Minas Gerais, Belo Horizonte, Brazil”, “Department of Biochemistry and Immunology, Institute of Biological Sciences, Federal University of Minas Gerais, Belo Horizonte, Brazil”, “Macrophage and Monocyte Biology Laboratory, Department of Pathology, Institute of Biological Sciences, Federal University of Minas Gerais, Belo Horizonte, Brazil” and “Department of Physiology and Biophysics, Institute of Biological Sciences, Federal University of Minas Gerais, Belo Horizonte, Brazil”, it should be “Center for Gastrointestinal Biology, Departamento de Morfologia, Instituto de Ciências Biológicas, Universidade Federal de Minas Gerais (UFMG), Belo Horizonte, Brazil”,“Department of Biochemistry and Immunology, Instituto de Ciências Biológicas, Universidade Federal de Minas Gerais (UFMG), Belo Horizonte, Brazil”, “Macrophage and Monocyte Biology Laboratory, Department of Pathology, Instituto de Ciências Biológicas, Universidade Federal de Minas Gerais (UFMG), Belo Horizonte, Brazil” and “Department of Physiology and Biophysics, Instituto de Ciências Biológicas, Universidade Federal de Minas Gerais (UFMG), Belo Horizonte, Brazil”.

The authors apologize for this error and state that this does not change the scientific conclusions of the article in any way. The original article has been updated.

## Publisher’s note

All claims expressed in this article are solely those of the authors and do not necessarily represent those of their affiliated organizations, or those of the publisher, the editors and the reviewers. Any product that may be evaluated in this article, or claim that may be made by its manufacturer, is not guaranteed or endorsed by the publisher.

