# Supplementary material for: Susceptibility to Infections During Acute Liver Injury Depends on Transient Disruption of Liver Macrophage Niche
Source: Front Immunol. 2022 Jul 28;13:892114. doi: 10.3389/fimmu.2022.892114 (PMC9368782; doi:10.3389/fimmu.2022.892114)
Supplement: Supplementary file 1 [file DataSheet_1.docx]

Supplementary Material

# Supplementary Figures and Tables

##
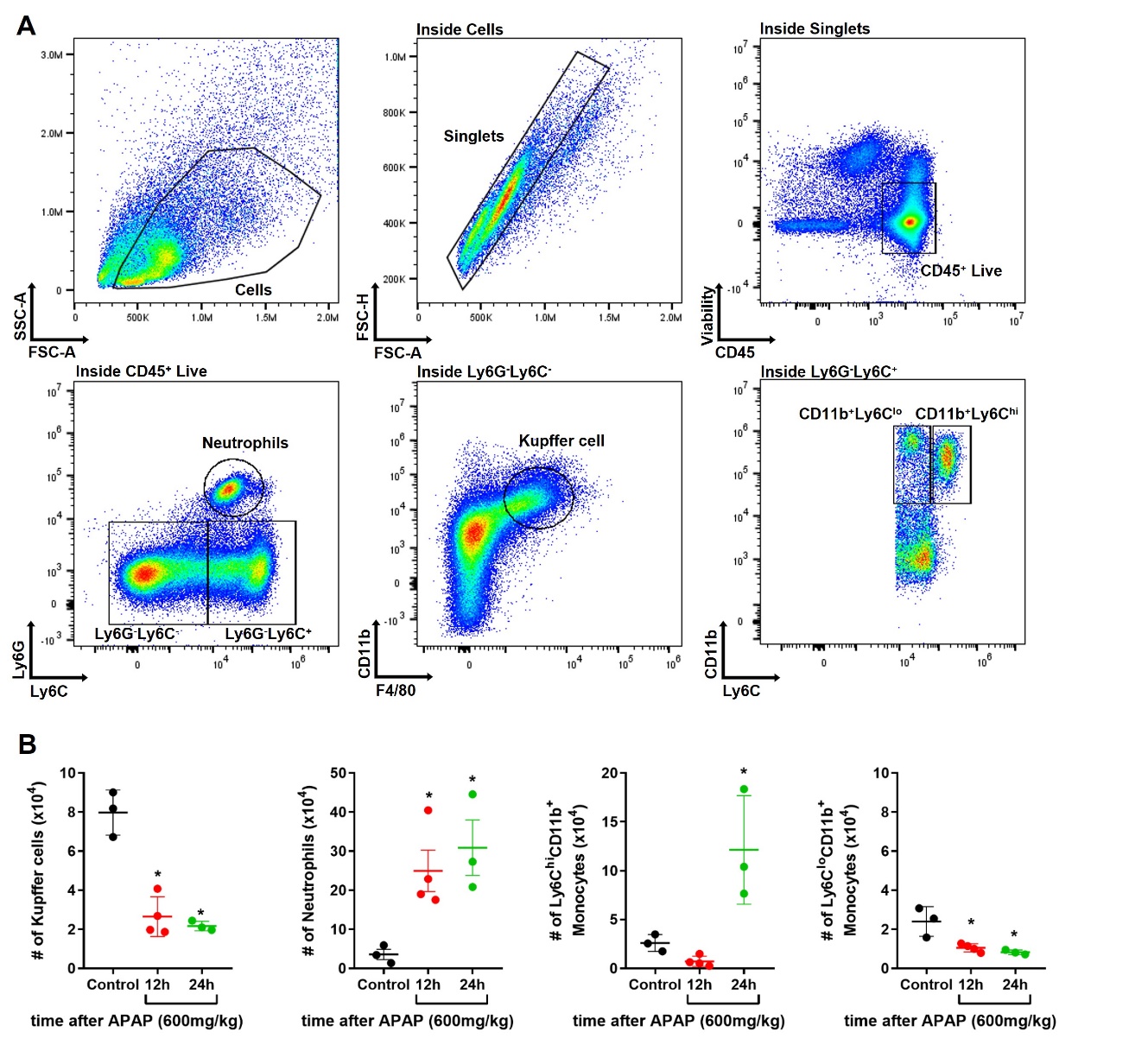
Supplementary Figures

**Supplementary Figure 1.** Acute liver inflammation induced by APAP overdose promotes tissue damage and alterations in myeloid cell numbers. **(A)** Gating strategy used to analyze the myeloid cell niche in the liver of control and injured mice. **(B)** Absolute number (#) of different populations of myeloid cell niche adjusted to total live non-parenchymal liver cells during the acute phase of liver inflammation obtained by Flow cytometry; n = 3-5 mice per group; Representative of 3 independent experiments. Data are presented as the mean ± SEM. * indicates a statistical difference between the marked and control groups using one-way ANOVA and Tukey post-test **(B)** (* = p < 0.05).


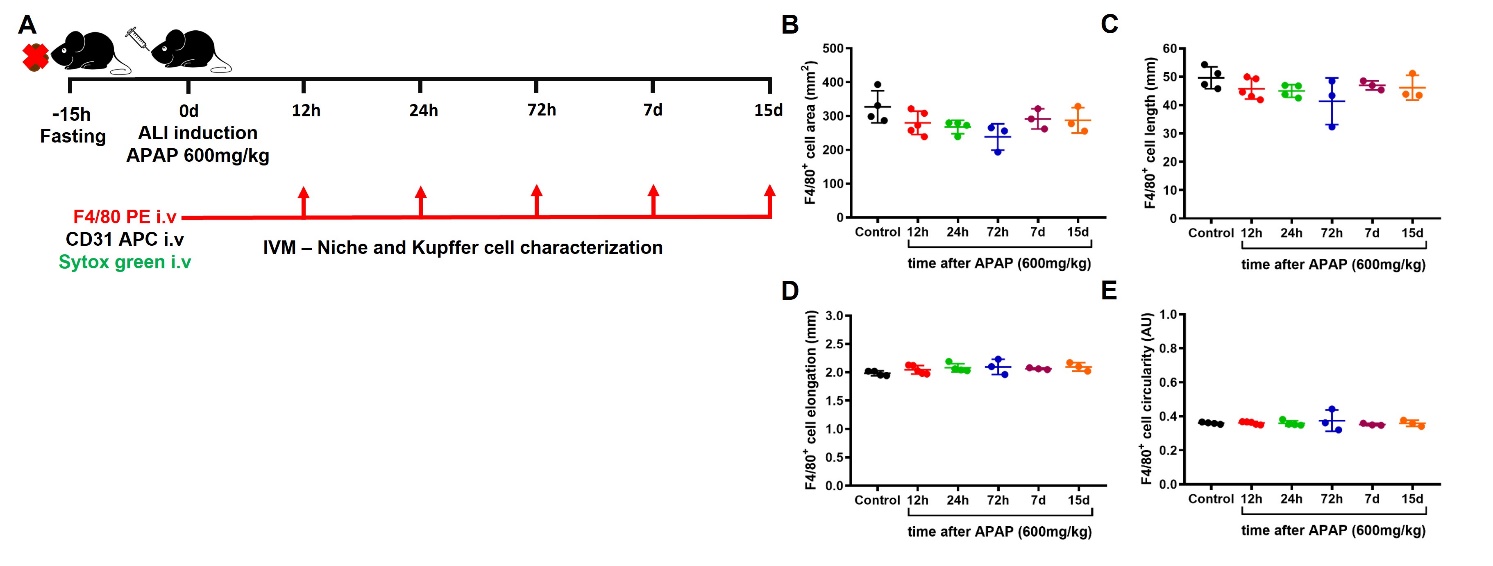


**Supplementary Figure 2.** Characterization of morphological parameters of Kupffer cells during ALI time course. **(A)** Scheme for Acute Liver Injury (ALI) induced by APAP overdose and intravenous injection of anti-F4/80 PE, anti-CD31 APC antibodies 20 minutes before surgery, intravenous injection of Sytox gree before anesthesia and Intravital microscopy assay. Definition of morphological aspects of Kupffer cells during ALI time-course including cell área **(B)**, cell length **(C)**, cell elongation **(D),** and cell circularity **(E)**. n ≥ 3 mice per group and 10 images per mice. Data are presented as the mean ± SEM.


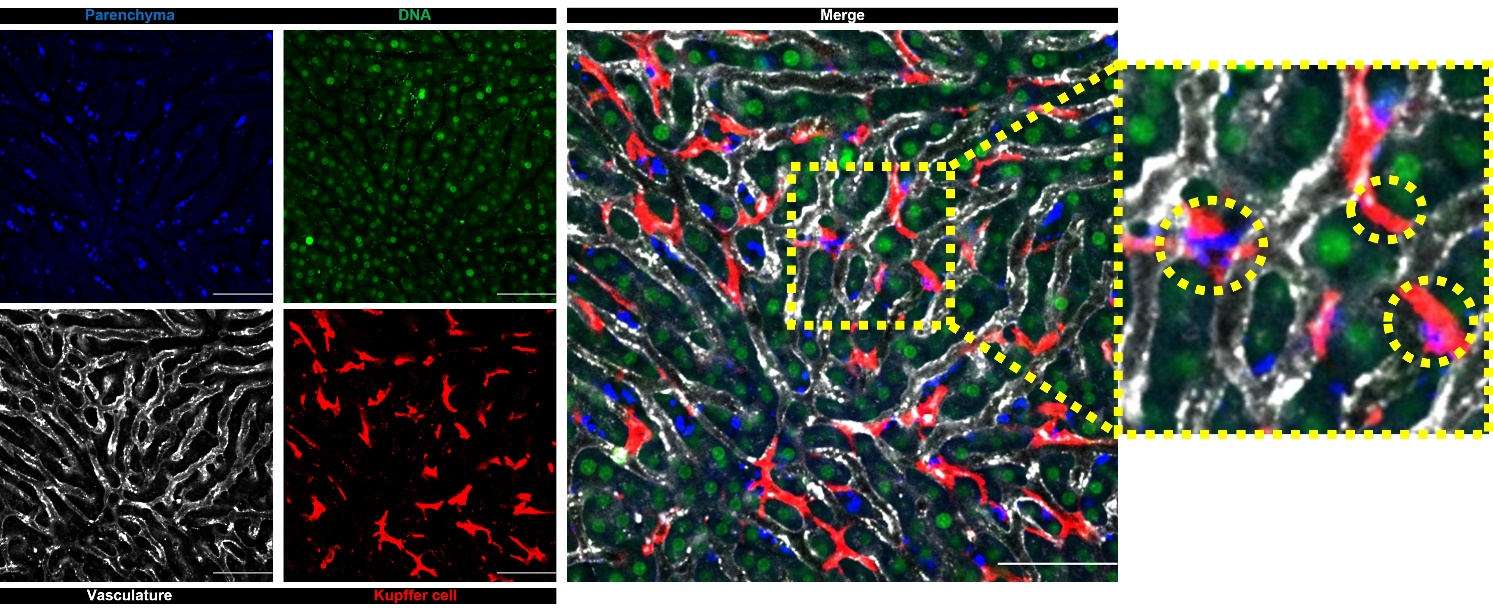


**Supplementary Figure 3.** Kupffer cells projections for outside the vasculature in a healthy mice liver. Representative intravital microscopy (IVM) images acquired from control mice to characterize Kupffer cell positioning inside the vasculature with projections (highlighted in yellow) to parenchymal space; Parenchyma is represented by liver auto-fluorescence; Vasculature is stained by anti-CD31 PE antibody; Kupffer cell is stained by anti-F4/80 APC; DNA is stained Sytox green. Scale bar = 50 µm.

**
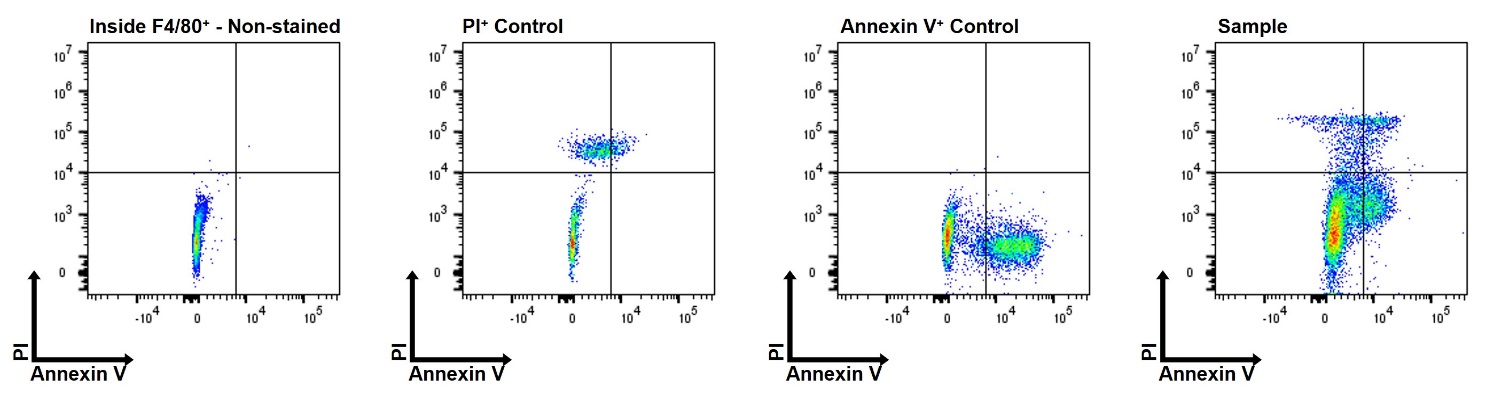
Supplementary Figure 4.** Gate strategy for Annexin V and Propidium Iodide (PI) staining inside F4/80^+^ cells.


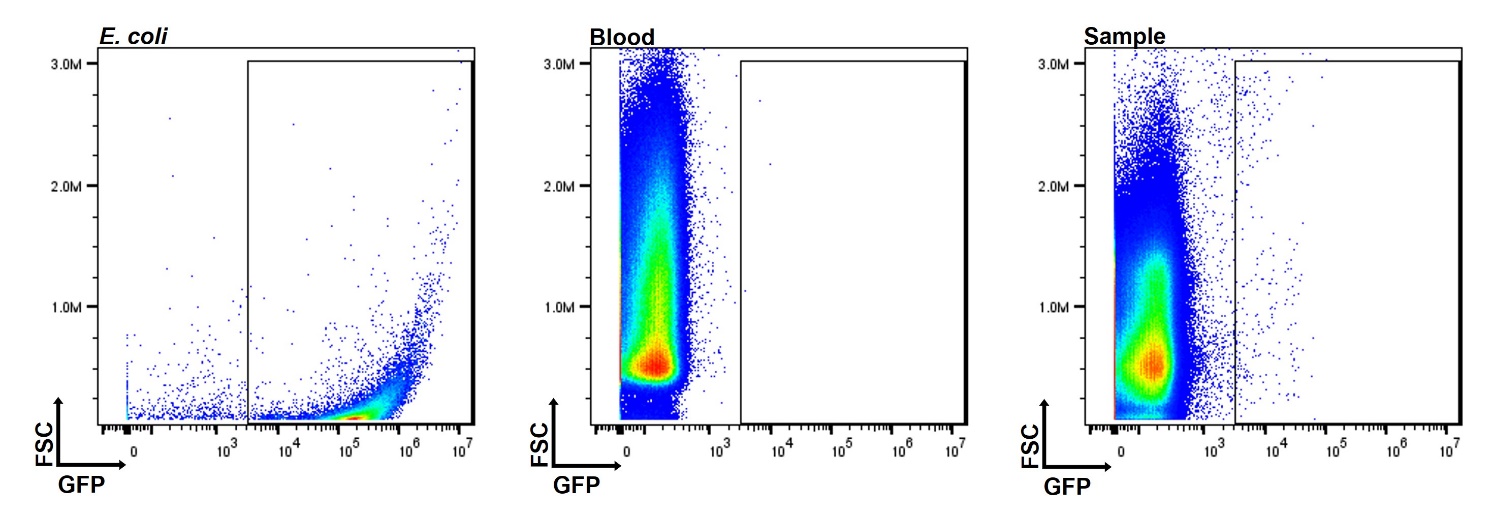


**Supplementary Figure 5.** Gating strategy used for bacterial events quantification on blood samples.


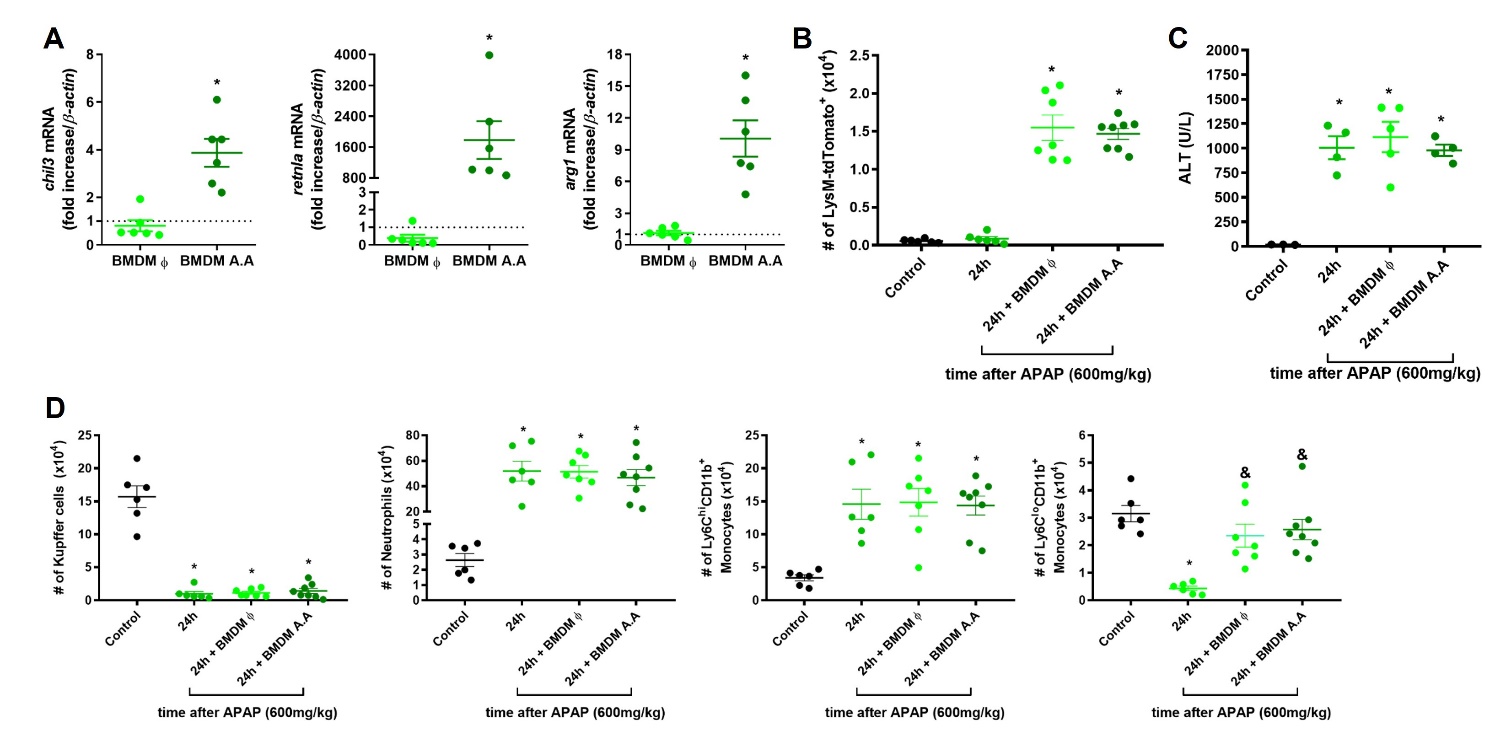
**Supplementary Figure 6.** Bone marrow-derived macrophages activation status and influence on liver myeloid cell compartment. **(A)** mRNA expression of BMDM A.A markers *chil3* (YM1), *retnla* (FIZZ1), and *arg1* (Arginase 1) - measured by real-time PCR and expressed by fold increase to *β-actin*); n ≥ 5 mice per group; representative of 4 experiments. **(B)** Absolute number (#, right panel) of LysM^cre/+^Rosa26^tdTomato/+^ cells adjusted to total live non-parenchymal liver cells during the acute phase of liver inflammation obtained by Flow cytometry; n ≥ 5 mice; representative of 2 independent experiments. (**C)** Measurement of Liver injury by serum levels of Alanine aminotransferase (ALT); n ≥ 4 mice per group. **(D)** Evaluation of myeloid cells populations after BMDM cell therapy by flow cytometry showing the absolute numbers of myeloid cell subtypes adjusted to total live non-parenchymal liver cells during the acute phase of liver inflammation obtained by Flow cytometry; n ≥ 5 mice; representative of 2 independent experiments. Data are presented as the mean ± SEM. * indicates a statistical difference between the marked and control groups using one-way ANOVA followed and Tukey post-test (* = p < 0.05). & indicate a statistical difference between the marked and 24h group (not treated with BMDM) using one-way ANOVA followed and Tukey post-test (* = p < 0.05).
